# Measurement Uncertainty of Surface Temperature Distributions for Laser Powder Bed Fusion Processes

**DOI:** 10.6028/jres.126.013

**Published:** 2021-08-10

**Authors:** David C. Deisenroth, Sergey Mekhontsev, Brandon Lane, Leonard Hanssen, Ivan Zhirnov, Vladimir Khromchenko, Steven Grantham, Daniel Cardenas-Garcia, Alkan Donmez

**Affiliations:** 1 National Institute of Standards and Technology, Gaithersburg, MD 20899, USA; 2 Karlstad University, 651 88 Karlstad, Sweden; 3 Centro Nacional de Metrología, Carretera a Los Cués, Municipio El Marqués, Querétaro C.P. 76246, México

**Keywords:** additive manufacturing, emissivity, measurement uncertainty, powder bed fusion, reflectometry, temperature, thermography

## Abstract

This paper describes advances in measuring the characteristic spatial distribution of surface temperature and emissivity during laser-metal interaction under conditions relevant for laser powder bed fusion (LPBF) additive manufacturing processes. Detailed descriptions of the measurement process, results, and approaches to determining uncertainties are provided. Measurement uncertainties have complex dependencies on multiple process parameters, so the methodology is demonstrated on one set of process parameters and one material. Well-established literature values for high-purity nickel solidification temperature and emissivity at the solidification temperature were used to evaluate the predicted uncertainty of the measurements. The standard temperature measurement uncertainty is found to be approximately 0.9% of the absolute temperature (16 AC), and the standard relative emissivity measurement uncertainty is found to be approximately 8% at the solidification point of high-purity nickel, both of which are satisfactory.

This paper also outlines several potential sources of test uncertainties, which may require additional experimental evaluation. The largest of these are the metal vapor and ejecta that are produced as process by-products, which can potentially affect the imaging quality, reflectometry results, and thermal signature of the process, while also affecting the process of laser power delivery. Furthermore, the current paper focuses strictly on the uncertainties of the emissivity and temperature measurement approach and therefore does not detail a variety of uncertainties associated with experimental controls that must be evaluated for future generation of reference data.

## 1 Introduction: Thermography of the Laser Power Bed Fusion Process

### 1.1 Relevant Features of the Laser-Based Additive Manufacturing Process

Laser power bed fusion (LPBF) technology involves selective layer-wise consolidation of metal powder by a high-intensity laser beam moving across the powder bed. The metal powder is melted by the high-intensity laser, and then it solidifies as a joined bead in the wake of the moving laser beam. Multiple parameters of the laser-induced melting process affect the quality of the solidified part. Reliable fabrication of functional parts involves optimal design of the process, as well as monitoring and control of the production process. LPBF is a relatively mature technology, but due to the complexity of the process, the choice and optimization of its parameters are still largely empirical, while a significant effort goes toward better understanding of the laser-melting process.

Metal LPBF is a highly dynamic thermal process caused by the fast-moving (on the order of 1 m/s) laser melting the metal. In terms of temperature distribution, the LPBF process is generally characterized by heating and cooling rates in the range of 1 K/µs to 40 K/µs and temperature gradients of approximately 5 K/µm to 20 K/µm [1], with peak radiance temperatures reaching 3 500 K and higher. The typical spatial scale is on the order of 25 µm to 500 µm, with the average powder size on the order of 30 µm, the laser spot size close to 80 µm, and the melt pool width on the order of 120 µm and length on the order of 600 μm to 800 µm for single-scan tracks [2], or 1 mm to 10 mm for back-and-forth hatch scanning in a three-dimensional (3D) build. Monitoring of temperatures across each layer has paramount importance in controlling (and potentially stabilizing) the process.

In addition to these high gradients and heating and cooling rates, molten metal convection and evaporation of the metal, including vapor recoil pressure, create surface features that in turn affect local emissivity (the unitless proportion of radiance emitted by the object normalized by the radiance emitted by an ideal blackbody at the same temperature, ranging from 0 to 1) and laser absorption patterns [3, 4]. Further complicating the scene, the evaporated and ejected by-products affect the thermal signature of the process through the addition of their emitted radiation, as well as by absorbing, reflecting, and refracting the light that is thermally emitted by the heat-affected zone (HAZ) of the build surface [5].

Multiphysics modeling and simulation efforts to predict the outcome of LPBF processes mostly use bare metal plates due to the complexity of the laser-powder interaction [6]. Temperature measurements *via* thermography are used to validate such models. Therefore, the effort reported in this paper was focused on thermography of single-scan tracks on a bare nickel alloy plate instead of a metal powder layer. With this approach, significant measurement noise in the presence of powder and its resulting dynamics was avoided.

### 1.2 Approaches to LPBF Thermography

Many commercial LPBF systems are equipped with melt pool monitoring instruments. These primarily use optical measurements to discern process instabilities from the radiant flux emitted by the melt pool in a certain spectral range or the relative morphology of the process area with intensity above a set threshold [7, 8].

Thermography may be used to determine temperature distributions across the field of view of the imager. Full spatial and temporal measurement of surface temperature is a complex and error-prone measurement. Therefore, most melt pool monitoring systems only acquire a signal proportional to radiance flux and then apply various signal or image analyses without attributing a temperature.

Traceability is a property of a measurement result whereby the result can be related to a reference through a documented unbroken chain of calibrations, each contributing to the measurement uncertainty [9]. Introduction of traceable process monitoring metrics potentially can enable part-to-part uniformity of the build process and resulting part quality, as well as better understanding of the phenomena behind failed builds.

Commercial single-band thermal imaging systems (detection in one relatively narrow range of wavelengths, or waveband) typically provide radiance temperature. The temperature can then be estimated using an assumed emissivity combined with the measured radiance temperature. This approach has value in documenting a particular build, because recorded radiance temperature is based on a calibration and reflects actual radiant intensities as observed. This can serve to establish equivalence of the building processes across different platforms.

A more metrological approach to melt pool thermography has shown appeal to the academic and research and development community involved in physics-based modeling and material development, where microstructure evolution is known to have a strong relation to the thermal history of the metal, including thermal gradients and cooling rates [10].

### 1.3 LPBF Thermography Configurations and Techniques

It is necessary to discuss two separate physical configurations of melt pool thermographic imagers, staring and coaxial, and two approaches to data analysis, single-band (spectral) thermometry and multiband approaches involving two or more bands. In a staring configuration, the imager has a stationary field of view that is fixed to an area that either fully or partially covers the build area. Staring imagery is practical only for a small area of the build space due to the limited depth of field, as well as the trade-offs among spatial resolution, covered area, and frame rate. At the same time, it offers the advantage of having an independent optical system, giving flexibility in spectral range and collection angle. Also, this technique naturally provides a record of the temperature evolution of a particular region of build space [11].

Coaxial techniques involve the use of a beam splitter in the path of the processing laser; the beam splitter is transparent for the laser, but it diverts light at a wavelength of interest to an imager. This causes the imager’s field of view to “follow” the laser along the same optical path. The resulting melt pool images appear nearly stationary, which simplifies image analysis and limits motion blur, which can be a issue with staring configurations. Another advantage is efficiency of the data acquisition since only the area in the vicinity of the melt pool is observed. This enables the frame rate of measurements to be increased as a trade-off for reducing the necessary image size, or reducing the overall volume of the data. Complications include sharing multiple optical elements with the process laser, limiting accessible spectral range or forcing some trade-offs on the optics, and limited numerical aperture [12].

Another option is two-color (ratio) thermometry, where the target temperature is calculated from the ratio of relative intensity of light emitted at different wavelengths [1, 13], which involves an assumption regarding the spectral characteristics of the target emissivity at these wavelengths. If emissivity is assumed to have “graybody” spectral characteristics (i.e., independent of wavelength), then a calibration with a blackbody source or a source with known spectral radiance is sufficient, as in Ref. [1]. However, the calibration is more often performed using the same measurement target and references a single point where temperature is known (using a thermocouple or a phase transition point, as in Ref. [13]). This approach has a strength in being resilient to emissivity changes or optical path losses, as long as they happen in a way that affects measurement at both wavelengths equally. Unfortunately, there are limited opportunities to establish the uncertainty of such a measurement, unless reference data are available to validate the measurement. The purpose of the current study was to develop a temperature measurement capability as a step toward generating reference data.

### 1.4 Determining the Distribution of the Surface Temperature

To establish traceable radiance-based temperature measurements, we selected the only first principle approach that is applicable. The approach consists of (1) direct measurement of spectral radiance of the HAZ by comparison with a radiance standard, (2) indirect measurement of spectral emissivity by surrounding the sample with a hemispherical illumination source and comparing reflectance measurements with a calibrated standard, and (3) calculation of the surface temperature distribution. We will describe this measurement methodology in a more rigorous way after establishing necessary nomenclature and referring to the known optical relations and physical laws, but the general approach to measurement of emissivity and radiance temperature has been realized and validated at the National Institute of Standards and Technology (NIST) earlier [14]. For the remainder of Sec. 1.4, we will discuss the unusual characteristics of the LPBF environment, which is much different from the one normally encountered in the radiometric systems used to generate reference data. The relatively small dimensions of the HAZ (with typical size of on the order of 0.5 mm × 1 mm) and the dynamics of the process necessitate use of imaging systems (or focal plane array [FPA] detectors) that enable retrieval of spatial as well as temporal data. Compared to the single-element optical detectors normally used in applied thermometry or instrument calibration, thermal imagers are much more complicated sensors with relatively large imperfections and uncertainties.

Use of a relatively wide spectral band for measurements (~40 nm wide at half of the peak intensity, or “half-height”) is quite common in order to acquire enough signal at the lower temperatures. This forces us to deal with so-called “band-limited” calculations, rather than using simpler, monochromatic or narrowband approximations. In this paper, “band-limited” indicates a spectrally varying value (*e.g.*, spectral radiance) that is integrated over some bandwidth, which typically defines the sensitive bandwidth of the sensor or imager.

The last and perhaps the most drastic difference in the laser-melting process lies in the fact that all optical measurements are performed in the environment that contains process by-products (such as hot metal vapor, metal condensate, and ejecta [3]). These by-products are formed at the laser-metal interaction spot and can potentially significantly emit, absorb, reflect, and refract the measured radiant emission. The relative contribution of the by-products can depend on laser power and velocity, metal properties, oxygen content, velocity of the shield gas, and other factors. The effects of process by-products on thermography are related to test uncertainty because they change based on processing conditions and are therefore not addressed in this paper. As will be described in the later sections of this paper, correcting for process by-products effects may increase the accuracy of thermography of a laser-induced melt pool.

### 1.5 Thermographic Data of Interest

While the experimental setup enables generation of several types of reference data relevant to the LPBF process, the scope of this paper is limited to thermographic data, which can involve the following measurands.

#### 1.5.1 Local Band-Limited Radiance

A thermally calibrated imager can directly provide a quantified signal (associated with each pixel) in the form of an image, where the signal is nominally proportional to the band-limited radiant flux on each pixel. With a thermal calibration, this signal can be converted to radiance temperature. The spatial distribution obtained from thermographic images of band-limited radiance or radiance temperature is a directly observable data product of the process thermography.

Band-limited radiance or radiance temperature can be measured and presented across much of the temperature range expected in an LPBF melt pool and HAZ, which is typically around 1 000 °C up to approximately 3 000 °C. Though individual measurements are limited to a specific range, radiance or radiance temperature data can be provided as a composite of multiple measurements at different integration times (or shutter speeds [SSs]).

#### 1.5.2 Local Band-Limited Effective Directional Emissivity

The effective emissivity, determined from a hemispherical-directional reflectance factor (HDRF), is obtained by illuminating the surface with a near-uniform hemispherical source (constant intensity over every angle), and measuring the reflected intensity in one direction. Here “effective” signifies the fact that it is not an intrinsic optical property of the material, but a result of a combination of several factors including varying shape, composition, and presence of vapor/ejecta. Furthermore, this effective directional emissivity is only relatable to emissivity at the direction and wavelength at which HDRF is measured.

This type of data can be obtained only for a partial range of sample temperatures from ambient to an upper limit determined by the intensity of the illumination of the reflectometer. It should also be noted that this directional effective emissivity is not the same as total hemispherical emissivity used in the Stefan-Boltzmann law for radiation heat transfer, although they are related, as discussed in further detail in Ref. [15]. The local band-limited effective directional emissivity will be referred to simply as “emissivity” for brevity in the remainder of this paper.

#### 1.5.3 Surface Temperature

This is a derived measurand, with values calculated from band-limited radiance (Sec. 1.5.1) and the effective directional emissivity (Sec. 1.5.2). A goal of thermography is to measure the surface temperature by evaluation of an approximate derived value, which is often called “true temperature.” This paper will assume the former is equivalent to the latter and will use these terms interchangeably.

### 1.6 Experimental Setup

The thermography experiments reported here were performed in the NIST Additive Manufacturing Metrology Testbed (AMMT) [16, 17]. The AMMT is a custom LPBF research platform that is designed to be highly configurable for measurement of all aspects of the LPBF process. The AMMT includes a removable carriage that contains the build-well and a large metrology-well, both of which may be moved laterally within the large build chamber. The laser is a Yb-doped fiber laser with emission wavelength of 1 070 nm. Laser power delivery can be adjusted from 20 W to more than 400 W, with a 4σ diameter spot size (where *D*4σ represents the diameter within which approximately 95% of the Gaussian laser power profile is contained); the spot size is adjustable from 45 µm to more than 200 µm. The laser spot can be scanned with velocities of up to around 4 000 mm/s while maintaining full control of the laser scan path [16].

**Fig. 1:**
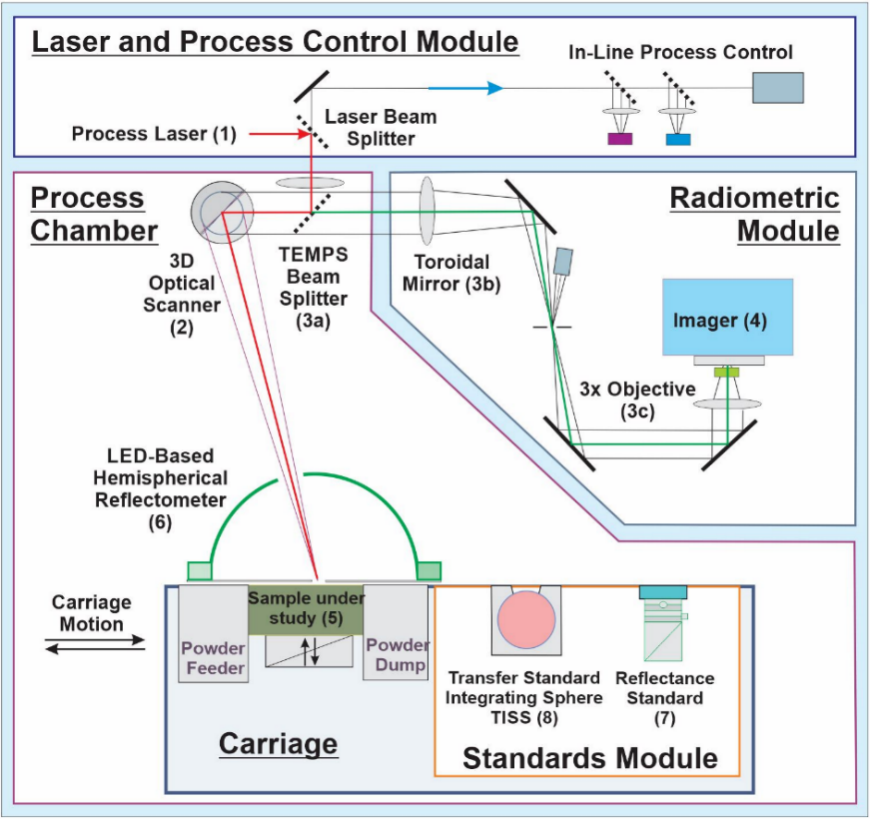
Simplified schematic of the AMMT. LED = light-emitting diode.

The relevant elements of the test bed, as shown in Fig. 1, include (1) a high-power fiber laser system emitting at 1 070 nm; (2) an optical scanner (galvanometer), used to direct the process laser spot; (3a—3c) a beam splitter and other optical components enabling coaxial melt pool imaging configuration; (4) an imager, and (5) the sample under study, which can be accurately positioned and aligned with the object plane of the coaxial laser/imager optical path, and which is surrounded by an environmental enclosure with a shield gas flow. Additional metrology equipment, which is referred to as the TEMPS system (temperature and emissivity of melts, powders, and solids), includes a hemispherical reflectometer (6), the reflectance standard (7), and a radiance temperature transfer integrating sphere source (8).

## 2 Nomenclature and Measurement Equations

### 2.1 Nomenclature

Before proceeding with the analysis, it is useful to briefly discuss the terminology.

***Radiant flux***, ***Φ***, is the power emitted or received in the form of optical radiation (measured in W). It should be noted that in this context, “flux” does not indicate a quantity divided by area.

***Spectral radiant flux***, ***Φ****_λ_*, is the ratio of the radiant flux taken over a spectral differential element *dλ* containing the wavelength *λ* to that interval:

**Table:** 

	$\Phi_{\lambda }=\frac{d\Phi (\lambda )}{d\lambda }\;\left[\frac{W}{m}\right]$	(1)

The subscript *λ* indicates the spectral radiant flux is a function of wavelength, *λ*.

***Spectral radiant intensity***, *I_λ_*, is the proportion of the differential element of radiant flux $d\Phi_{\lambda }$ leaving the source and propagating through the differential element of solid angle *dΩ* containing the given direction:

**Table:** 

	$I_{\lambda }=\frac{d\Phi_{\lambda }}{d\Omega }\;\left[\frac{W}{m∙sr}\right]$	(2)

***Radiance***, ***L***, is the quantity defined as

**Table:** 

	$L=\frac{d\Phi }{dA∙cos\theta ∙d\Omega }\;\left[\frac{W}{m^{2}sr}\right]$	(3)

where *d****Φ*** is the radiant flux transmitted by an elementary beam passing through the given point and propagating in the solid angle *dΩ* containing the given direction; *dA* is the area of a section of that beam containing the given point; and *θ* is the angle between the normal to that section and the direction of the beam.

***Spectral radiance***, ***L_λ_***, is the quantity defined as

**Table:** 

	$L_{\lambda }=\frac{d\Phi (\lambda )}{dA∙cos\theta ∙d\Omega ∙d\lambda }\;\left[\frac{W}{m^{3}sr}\right]$	(4)

where *d****Φ***(*λ*) is the radiant flux taken over an elementary spectral interval *dλ* containing the wavelength *λ*, transmitted by an elementary beam passing through the given point and propagating in the solid angle *dΩ* containing the given direction; *dA* is the area of a section of that beam containing the given point; and *θ* is the angle between the normal to that section and the direction of the beam. Spectral radiance is considered to be the most fundamental and basic unit, because it is directly related to observable radiometric values and the resulting value of Planck’s law.

***Total radiance*** is related to the ***spectral radiance*** as

**Table:** 

	$L=\int_{0}^{\infty }L_{\lambda }d\lambda$	(5)

Additionally, total radiance and spectral radiance can be indicated as directionally dependent, *L(****ω****)* or *L_λ_(****ω****)*, where ***ω*** specifies the direction of observation.

***Spectral responsivity of the sensor***, *r^FPA^(λ)*, is the spectrally dependent relative output signal response from a sensor (most often an FPA detector in this paper) when exposed to direct monochromatic input. It is typically expressed as a unitless function of wavelength between 0% and 100%, and it is solely a characteristic of the sensor and excludes other optical elements in a system.

***Spectral responsivity of the optical system***, *r(λ)*, is the relative output signal response from a sensor, including all optical system components, when exposed to direct monochromatic input. The value of *r(λ)* is typically expressed as a unitless function of wavelength between 0% and 100%. Here, *r(λ)* is defined as a combination of both the spectral responsivity of the sensor and spectral reflective or transmissive characteristics of all optical components in the path of the sensor.

***Band-limited radiance***, *L_BL_*, is the proportion of radiance that is detectable by a given sensor within its usefully responsive waveband (*λ_1_→λ_2_*), and is defined as

**Table:** 

	$L_{BL}=\frac{\int_{\lambda_{1}}^{\lambda_{2}}r(\lambda )L_{\lambda }d\lambda }{\int_{\lambda_{1}}^{\lambda_{2}}r(\lambda )d\lambda }$	(6)

***A perfect blackbody*** is an idealized radiating source that completely absorbs all radiation incident upon it at all wavelengths and incident angles and emits the maximum amount of thermal radiation at a given wavelength and temperature in comparison with any other thermally radiating bodies. Its spectral radiance, $L_{BB}\left(\lambda ,T\right)$, is described by a **Planck function**, $Pl\left(\lambda ,T\right)$, such that

**Table:** 

	$Pl\left(\lambda ,T\right)\equiv \frac{c_{1}}{{{\pi \;n}^{2}\lambda }^{5}\left[\exp(c_{2}/(n\lambda T))-1\right]}$	(7)

where $L_{BB}\left(\lambda ,T\right)=\;Pl\left(\lambda ,T\right)$, *T* is blackbody temperature [K], *λ* is wavelength [m], and *n* is the index of refraction of the environment, and where *c*_1_ = 3.74177153 × 10^−16^ W∙m^2^ and *c*_2_ = 1.4387770 × 10^−2^ m∙K are the first and the second radiation constants, respectively [18, 19]. In further treatment, we will omit the refractive index of the air because it is close to unity (*n* = 1.000293 in the spectral range of interest), and we will also use a modified first radiation constant, *c*_1_*_L_= c*_1_*/π*. The refractive index of argon, which exists in a small fraction of the optical path, is also negligibly different from unity.

***Local directional spectral emissivity***,$\;\varepsilon \left(\lambda ,\;\omega ,T\right)$, is the ratio of spectral radiance of the emitting object at a specific wavelength *λ*, direction ω, and temperature *T* to that of a perfect blackbody at the same temperature *T* and wavelength *λ*,

**Table:** 

	$\varepsilon \left(\lambda ,\;\omega ,T\right)=L_{\lambda }(\lambda ,\;\omega ,T)/Pl(\lambda ,T)$	(8)

where the vector ***ω*** specifies the direction of observation, often given in polar *θ* and azimuthal *ϕ* angles in spherical coordinates. Also note that since a perfect blackbody emits isotropically, there is no directional term in the denominator.

***Spectral reflectance ρ****(λ)*, ***spectral transmittance τ****(λ),* ***and spectral absorptance*** *𝜶(λ)* are used in optical engineering to describe optical properties of the materials. These quantities are described as the ratio of the reflected, transmitted, and absorbed radiant flux to the monochromatic incident radiant flux at the wavelength *λ*. Due to energy conservation law,

**Table:** 

	***ρ****(λ)+ ****τ****(λ)+ 𝜶(λ) = 1*	(9)

For an opaque surface, according to Kirchhoff’s law and reciprocity principle,

**Table:** 

	***ε**** (λ, ****ω****) = 𝜶(λ, ****ω****_i_)*	(10)

where the subscript *i* indicates the incident angle of radiation on the surface, and ***ω*** *= −****ω****_i_* is the direction of observation, presumed to be directly opposite the incident direction with respect to the surface normal. Application of the energy conservation law gives for opaque material (*τ(λ) =* 0):

**Table:** 

	***ε**** (λ, ****ω****)= 1 − ****ρ****(λ, ****ω****_i_)*	(11)

***A calibration blackbody*** is a physical artifact designed to approximate characteristics of a perfect blackbody, and its performance is described by two parameters, reference temperature, *T*_ref_, and effective spectral emissivity, ***ε***_eff_, where the choice of reference temperature is user selectable and may or may not equate to a real blackbody temperature.

***Band-limited calibration source*** is a physical artifact that emits light over a limited spectral range and provides radiance temperature calibrated for that spectral range against another band-limited or blackbody emitter source. For example, the transfer integrating sphere source (TISS 850) is discussed in Sec. 4.2.4.

***Radiance temperature*** (also known as “brightness temperature” or “apparent temperature”), *T*_rad_, is the temperature of a perfect blackbody that has the same spectral radiance as a real object of interest:

**Table:** 

	$L_{\lambda }\left(\omega ,\lambda ,T\right)=Pl(\lambda ,T_{rad})$	(12)

Note that the left side of Eq. (12) represents the spectral-directional radiance of a real object, and the right side is for an idealized perfect blackbody. Radiance temperature is not a physical, thermodynamic temperature, but a conceptual value that is characteristic of the radiating object.

***Linear detector*** is a radiometric sensor, either a single detector or pixel within an array/imager, that outputs a signal that is linearly proportional to the incident radiant flux on the detector:

**Table:** 

	$S\;\propto \Phi =\int_{0}^{\infty }r\left(\lambda \right)\varepsilon \left(\lambda ,\omega \right)Pl\left(\lambda ,T\right)d\lambda$	(13)

When responsivity of the detector, $r\left(\lambda \right)$, depends on the flux value, a nonlinearity correction can be implemented by mapping responsivity change. This is done by varying the flux and recording the signal, and then using this data set to linearize the signal during subsequent measurements (refer to Sec. 3.3.4.3 on linearization).

***Lambertian reflector*** (“diffuse”) has reflected radiance that is invariant with regard to the viewing angle or the incident angle of an illuminating source.

***Spectral directional-hemispherical reflectance*** (spectral DHR), ***ρ****(λ, θ)*, is defined as the ratio of spectral radiant flux integrated over all hemispherical solid angles (equivalent to 2π sr) to the incident spectral radiant flux on an element of a surface irradiated from a specific direction ***ω****_i_*. For isotropic objects, where there is no azimuthal *ϕ* variation on reflectance, incident angle ***ω****_i_* is equal to *θ* with respect to the surface normal.

***Effective spectral emissivity*** is a measured or assumed spectrally dependent emissivity, nominally related to the true material emissivity of the measured surface, but used in practice for evaluating surface temperature from radiance temperature measurements. Effective spectral emissivity convolves various real-world effects such as surface finish effects, complex surface topography or multiple surface reflections, or locally varying surface temperature over the area of interest.

***Background radiation effects*** create additional measured radiance stemming from sources other than the radiating object of interest. These are typically radiating sources within the environment that reflect off the measured object of interest, creating surplus measured signal. This additional radiant flux can also be initiated from one part of the object and reflect off another part. The apparent (experimentally observable) spectral radiance can be expressed in general as:

**Table:** 

	***L****_APP_(λ,****ω***,*T)* ***= L****_SELF_ (λ,****ω***,*T) +* ***L****_REFL_(λ,****ω***,*T_bg_)*	(14)

where *L*_SELF_ indicates the self-emitting radiance of the object of interest, and *L*_REFL_ indicates the contribution caused by reflected background radiation at (assumed) temperature *T*_bg_.

If the surrounding background radiation is uniform, isotropic, and can be characterized by *T*_bg_, and our target has known effective spectral emissivity ε(λ) and temperature *T*, and it possesses Lambertian reflector characteristics, then

**Table:** 

	$L_{APP}(\lambda ,T)=\varepsilon (\lambda )\cdot Pl(\lambda ,T)+(1-\varepsilon (\lambda ))\cdot Pl(T_{bg})$	(15)

### 2.2 Measurement Equations for Determination of Temperature and Emissivity Distributions

This section (Sec. 2.2) starts with a single-element simplified treatment of the measurement problem, followed by treatment of additional effects due to imager-based measurement.

#### 2.2.1 Representing the Emitting Object

The object of interest, viewed at a direction ***ω*** (assuming direction is constant across the object), can be characterized by the spatial distribution of spectral radiance ***L****_λ_(****x***,***ω****)*, where the vector ***x*** *= (x,y)* indicates a location on the object plane. This in turn can be expressed as a product of local spectral emissivity and the Planck function as calculated for the local temperature value *T(****x****)*:

**Table:** 

	$L_{\lambda }\left(x,\omega ,T\right)=\varepsilon \left(\lambda ,x,\omega \right)\cdot Pl(\lambda ,T(x))$	(16)

This equation allows one to calculate the spatial distribution of surface temperature T(x), if the locally variant radiance $L_{\lambda }\left(x,\omega ,T\right)\;$and spectral emissivity $\varepsilon \left(\lambda ,x,\omega \right)$ distributions are determined experimentally. With the experimental measurement apparatus of interest, all radiative properties are near-normal with less than 8° variation from the normal vector of each surface. Therefore, the directional dependence of each property will no longer be indicated by vector notation for the remainder of this paper.

#### 2.2.2 Temperature Measurement Equation for Idealized Case (Monochromatic System with Ideal Imaging and Infinitesimal Detector Size)

**Fig. 2:**
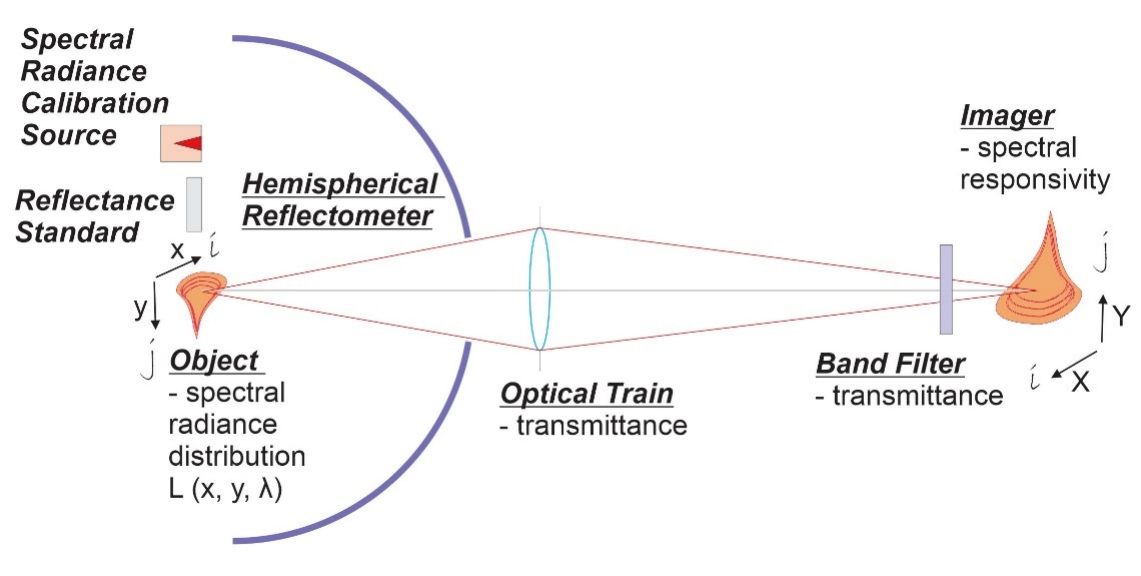
Simplified hemispherical reflectometer measurement setup.

To determine the spatial temperature distribution from Eq. (16), we need to measure spectral radiance and emissivity. This is realized using a hemispherical illumination source (also referred to as “dome” or “reflectometer”) based on an integrating-sphere reflectometer, which is then placed over the measurement object observed by the thermographic system, as shown in Fig. 2. The hemispherical illumination source provides measure of the HDRF (which is equivalent to DHR, first presented in Sec. 2.1 and discussed in detail in Sec. 3.2) by providing uniform illumination on the object by directionally integrating emission from multiple high-power light-emitting diodes (LEDs).

For spectral radiance measurements, we calibrated our system by pointing it to the standard source with known and spatially uniform radiance $Pl(\lambda_{0},T_{ref})$, where $T_{ref}$ stands for the apparent temperature of the reference source, and $S_{cal}$ is an imager signal during calibration. The calibration coefficient $C_{cal}$ at the wavelength of interest $\lambda_{0}$ can be determined from Eq. (15). Please note that in our current treatment, we use a single calibration for the whole sensor and treat any residual nonuniformity as uncertainty.

**Table:** 

	$S_{cal}\left(\lambda_{0},T_{ref}\right)=C_{cal}∙Pl(\lambda_{0},T_{ref})$	(17)

Since the calibration coefficient is now known, the spatial distribution of radiance $L(\lambda_{0},x,T_{samp})$ across the sample of interest can be determined from the measured signal $S_{samp}$:

**Table:** 

	$S_{samp}\left(\lambda_{0},X,T_{samp}\right)=C_{cal}∙L(\lambda_{0},x,T_{samp})$	(18)

where ***X*** is the location vector on the FPA corresponding to location vector ***x*** on the object plane.

#### 2.2.3 Emissivity Measurement Equation for Idealized Case (Monochromatic System with Ideal Imaging and Infinitesimal Detector Size)

To obtain the emissivity distribution, we can use Eq. (8) to Eq. (11) to establish the relation between reflectance and emissivity. The reciprocity principle allows for measurement of HDRF instead of DHR. The measurement methodology involves use of the hemispherical reflectometer (as shown in Fig. 2).

The radiant flux that is directionally reflected from the sample and incident on the detector is proportional to the linearized camera signal, as described in Eq. (13). The reflectance of an unknown sample can then be measured by comparing the signal received from an illuminated sample with the signal received from an illuminated absolute reflectance standard. The ratio of these two signals multiplied by the absolute reflectance of the standard determines the reflectance of the sample in the absence of any sample self-emission or background radiation. In this study, there was significant self-emission from the laser-induced melt pool, and so the signal received from the sample without illumination is subtracted from the signal received with illumination. In order to correct for any signal generated by the detector in the absence of incident radiant flux (also referred to as dark signal) or any stray background radiation, the detector signal received without illumination of the reflectance standard is also subtracted from the signal received with illumination of the reflectance standard.

Thus, in this treatment, the emissivity measurement is performed in four steps, with the measurement equation shown in Eq. (19). The laser-induced melt pool is first illuminated and imaged ($S_{samp}^{LED\;On}\left(\lambda_{0},x\right)$). The laser-induced melt pool is then imaged without LED illumination ($S_{samp}^{LED\;Off}\left(\lambda_{0},x\right)$). The reflectance standard is then recorded with ($S_{refl.st}^{LED\;on}\left(\lambda_{0},x\right)$) and without ($S_{refl.st}^{LED\;off}\left(\lambda_{0},x\right)$) LED illumination. The absolute reflectance of the standard is ($\rho_{ref.st}$).

**Table:** 

	$\varepsilon (\lambda_{o},x)=1-\rho_{ref.st}∙\frac{S_{samp}^{LED\;On}\left(\lambda_{0},x\right)-S_{samp}^{LED\;Off}\left(\lambda_{0},x\right)}{S_{refl.st}^{LED\;on}\left(\lambda_{0},x\right)-\;S_{refl.st}^{LED\;off}\left(\lambda_{0},x\right)}$	(19)

#### 2.2.4 Complete Calibration Equation

The practical measurement differs from the idealized approach described in Sec. 2.2.3 in many ways, with different effects that can be grouped by the following:

(1) spectral effects (*e.g.*, finite spectral width of the sensor system [non-monochromatic case]);
(2) spatial effects, caused by nonideal optics with finite resolution and scattering, finite size of the pixel, FPA nonuniformity, and pixel cross-talk); and
(3) systematic variability, such as temporal jitter of the optical path (due to nonideality of scanning mirrors) and sample surface variation, *etc*.

The signal of a single pixel, $S\left(i,j\right)$, of the FPA can be expressed as an integral of local spectral radiance of the object element $L_{\lambda }\left(x\right)$ multiplied by the spectral responsivity of the imaging system $r_{\lambda }$ and integrated over the finite band *λ1→λ2*, which encompasses *r_λ_*.

**Table:** 

	$S\left(i,j\right)=C_{cal}\int_{\lambda 1}^{\lambda 2}r_{\lambda }L_{\lambda }\left(x\right)d\lambda$	(20)

As introduced in Eq. (15), $C_{cal}$ is a calibration constant determined during system-level calibration against a calibration blackbody or appropriate band-limited source. In our case, the system responsivity, *r_λ_*, for all pixels can be expressed as follows, based on individual contributions of components in the system:

**Table:** 

	$r_{\lambda }=\tau_{\lambda }^{filt}‧\tau_{\lambda }^{opt}{‧r}_{\lambda }^{FPA}$	(21)

where $\tau_{\lambda }^{filt}$ is the relative spectral transmittance of the spectral band-pass filter, $\tau_{\lambda }^{opt}$is the relative spectral transmittance of the optical train, and $r_{\lambda }^{FPA}$ is the relative spectral responsivity of the FPA.

The measurement equation for signal $S\left(i,j\right)\;$obtained from FPA pixel *(i,j)* is as follows, accounting for spectral and spatial integration effects, but assuming perfect imaging and sensor:

**Table:** 

	$S\left(i,j\right)=\;C_{cal}\iint_{x\in i}^{\;}\int_{\lambda =0}^{\infty }\tau_{\lambda }^{filt}\tau_{\lambda }^{opt}r_{\lambda }^{FPA}\varepsilon \left(\lambda ,x\right)Pl[\lambda ,T(x)]d\lambda dxdx$	(22)

where spatial integration is performed over the source area optically conjugated with the pixel (i,j).

To account for the effects of imperfect imaging (optical blur, pixel cross-talk, blooming, out-of-field stray light), additional corrections are made to S(i,j) on a per-pixel basis, to form a new image $S^{*}(i,j)$. With these corrections, a full measurement equation for signal spatial-effect-corrected $S^{*}(i,j)\;$obtained from FPA pixel (i,j) looks as follows:

**Table:** 

	$S^{*}\left(i,j\right)=\;\eta \left(i,j\right)\;\zeta \left(i,j\right)\;S(i,j)$	(23)

where additional terms take into account two additional effects—FPA imperfections (such as cross-talk and blooming), characterized by function $\eta \left(i,j\right)$, and optics scattering (or blur), characterized by function $\zeta \left(i,j\right)$. Note that these functions or corrections depend on the specific image being measured, in conjunction with a measured term that characterizes the spatial effect, p(i,j), such that $\zeta \left(i,j\right)=f_{1}[S\left(i,j\right),p\left(i,j\right)]$ and $\eta \left(i,j\right)$ =$f_{2}[S\left(i,j\right),p(i,j)$].

The remainder of this paper is laid out as follows. Section 3 and Sec. 4 establish the basis of thermometry by treating each pixel as if it is observing a uniform field. Section 5 will address the spatial effects on, and uncertainties of, the FPA signal. Section 6 provides measurement results of emissivity and temperature of a laser-induced melt pool, along with their measurement uncertainties. As shown by Eq. (22), the spatially resolved emissivity, $\varepsilon \left(\lambda ,x\right)$, must be measured before temperature can determined. Therefore, the measurement methodology for emissivity and evaluation of its uncertainty will be discussed in Sec. 3.

## 3 Establishing Local Directional Effective Band-Limited Emissivity Distribution from Reflectometry

As discussed in the preceding sections, an “indirect” method of emissivity measurement is employed in this work. The laser-melting process is uniformly illuminated by a hemispherical illumination source, which is referred to as a reflectometer. The reflectometer uses a hemispherical-directional geometry, in which a ring of LEDs around the equator of the hemisphere is optically integrated within the reflectometer to provide uniform illumination. The laser-melting scene is then imaged directionally through imaging optics that are coaxial with the heating laser. The measurement is performed once with the LED illumination on, and once with the LED illumination off. This approach facilitates spatially resolved radiance and reflectance/emissivity measurement of an object such as a scanned melt pool by maintaining a stationary image of the melt pool in the camera field of view. The measurement equation for emissivity will be discussed in Sec. 3.1. It should be noted that for the analysis presented in Sec. 3, no compensation for spatial effects (optical blur, stray light, *etc*.) is performed. Nevertheless, Sec. 3 establishes a framework for uncertainty analysis, which will be used along with compensation for spatial effects in Sec. 5. The methods in this section (Sec. 3) are built upon the method described in our earlier paper in Ref. [20].

### 3.1 Measurement Equation

The emissivity measurement equation for a single pixel of the FPA, assuming uniform intensity across the pixel, is as follows:

**Table:** 

	$\varepsilon (\lambda_{0},T_{s})=1-C_{\rho }\rho_{ref}\frac{S_{samp}^{LED\;On}\left(\lambda_{0},T_{s}\right)-S_{samp}^{LED\;Off}\left(\lambda_{0},T_{s}\right)}{S_{ref}^{LED\;on}\left(\lambda_{0}\right)-\;S_{ref}^{LED\;off}\left(\lambda_{0}\right)}$	(24)

where *ε* is the normal (or 8° from normal) spectral emissivity of the sample, $\lambda_{0}$ is the wavelength of the detector (and the illumination source), $T_{s}$ is the sample temperature, and $\rho_{ref}$ is the reflectance of the calibrated reference standard. The linearized signal measured by a single pixel of the imager is denoted “*S.*” The signal linearization approach and its uncertainties will be discussed in Sec. 3.3.4.3. The superscripts “LED on” and “LED off” refer to signals obtained with and without LED illumination. The subscripts “Samp” and “ref” indicate when the object of measure is the sample and calibrated reflectance standard, respectively. The term $C_{\rho }$, which is nominally 1, is used as a correction factor for systematic biases. It also has an associated uncertainty, which is propagated into the uncertainty of $\varepsilon_{\lambda }$. The correction factor $C_{\rho }$ is comprised of multiple correction factors, which are associated with each source of bias, nonideality, and uncertainty associated with the reflectometer:

**Table:** 

	$C_{\rho }=C_{TU}C_{PL}C_{LED}C_{ARM}C_{AS}C_{BRDFS}C_{Sub}C_{S}$	(25)

where $C_{TU}$,$C_{PL},C_{LED},C_{ARM},C_{AS},C_{BRDFS},$ $C_{Sub},$ and $C_{S}$ are the factors associated with throughput uniformity, port losses, and high-angle losses, LED reproducibility, alignment of the reflectance standard (also referred to as the reference mirror), alignment of the sample, the sample bidirectional reflectance distribution function (BRDF), substitution error, and out-of-field scatter, respectively. Each one of these correction factors is discussed in Sec. 3.3. Section 3.2 will describe the important design considerations of the reflectometer used for these measurements.

### 3.2 Reflectometer Design

The design of the reflectometer is constrained by the size of the build chamber, the necessary gas flow provisions for laser-metal interaction, fabrication limitations, and the likelihood of damage to the internal reflective coating by laser reflection from the melting process. In the current case, a hemispherical reflectometer design is used instead of a full sphere in order to address the unique considerations of the LPBF environment while maintaining illumination performance. Use of a hemispherical integrating reflectometer (as opposed to full sphere reflectometer) has been applied for compact size and other optical considerations, but it has not yet been applied to temperature measurement of LPBF to the best of our knowledge [21].

The use of a hemisphere, instead of a full sphere, allowed for a small laser-entrance port area and sample port area, relative to the total integration area, while fitting within the height of the build chamber of the AMMT. An equivalent-height full integrating sphere would have had approximately double the ratio of the port area to integration area. Regarding coating damage, the hemispherical design allowed the integrating surface to have greater average distance from the laser processing. In a full sphere design, this would range from very close to the entrance port to double the distance at the maximum angle of interest. Irradiance (radiant flux received by a surface) is proportional to the square of distance from the emitter, and so the hemispherical design reduces the diffuse coating exposure to intense laser reflections on average by approximately a factor of two. Furthermore, the hemispherical geometry provided double the perimeter for LED illumination, also facilitating approximately double the intensity of illumination, which must be on the same order of magnitude as the process self-emission. A cross-section view of a computer-aided design model of the integrating hemisphere is shown in Fig. 3a. The fabricated base, hemisphere interior, and assembled integrating hemisphere within the build chamber are shown in Fig. 3b through Fig. 3d.

As shown, optical integration is facilitated by a diffuse, barium sulfate coating and with a specular, polished aluminum base electroplated with gold, which has excellent reflectivity at the wavelength of 850 nm, which is the most often used wavelength in this measurement system. The measured object, hemispherically and uniformly illuminated, is imaged through an elongated port on the top of the hemisphere, approximately 8° from vertical. The 8° offset of the port prevents retroreflection from a specular (or nearly specular) sample into the inline optics, reducing the possibility of incomplete and/or nonuniform illumination of the sample due to the detection port. Furthermore, in the case of the current application, reduced likelihood of retroreflection from the sample into the inline optics also reduces the possibility of damage to the filters or the imager from retroreflected high-power laser light. The elongated design of the laser port and sample port allows scans to be done in an area of approximately 3 mm × 20 mm with coaxial imaging of the laser-metal interaction scene.

Another design constraint unique to the LPBF environment is the inert gas atmosphere required for laser-melting to reduce detrimental oxidation [22]. Previous studies have shown that directional and inert shield gas flow is essential to facilitate continuous, consistent beam delivery by removing process by-products that can distort, scatter, and obstruct beam delivery [23, 24]. As shown in Fig. 3, Ar gas is pumped into the reflectometer through the laser port. The gas flow rate is typically approximately 30 L/min, which results in a vertically directed downward flow onto the sample with an average velocity of approximately 0.5 m/s. This provides some by-product removal stemming from the laser-metal interaction, though future measurements will incorporate an improved directional gas flow provision.

The thickness of the base of the dome acts as a baffle and results in an illumination angle of approximately 135°, as shown in Fig. 3. The LEDs are located as close as possible to the equator of the hemisphere. At this location, the base thickness has a beneficial baffle effect, and it prevents deleterious direct illumination of the sample by the LEDs, which would create excess, directionally dependent illumination outside the desired hemispherically uniform illumination. The base thickness, though, causes a loss of light in the remaining 45° of the hemisphere, which does not contribute to illumination. In practice, surface features reflecting light into the directional imaging path at an angle less than 22° from the horizontal are unlikely, but possible, and the associated contribution to surface reflectance/emissivity measurement uncertainty must be evaluated. Each source of error and uncertainty associated with the reflectometer-based measurement of emissivity will be described in Sec 3.3.

### 3.3 Measurement Uncertainty of Band-Limited Emissivity

Four categories of emissivity measurement uncertainty have been identified. The uncertainties associated with the variables of Eq. (24), including the uncertainties of the seven correction factors contained in Eq. (25), are grouped within these categories. The first category is nonuniformity and incomplete hemispherical illumination, which pertains to the nonideality of the reflectometer. The second incorporates the nonideality, misalignment, and uncertainty in reflective character of the reference mirror. The third incorporates the misalignment, nonideality, and relatively unknown reflective character of the sample. The fourth and final category is the nonideality of the directional imaging system, including the inline optics and imager.

**Table:** 

**(a)**		
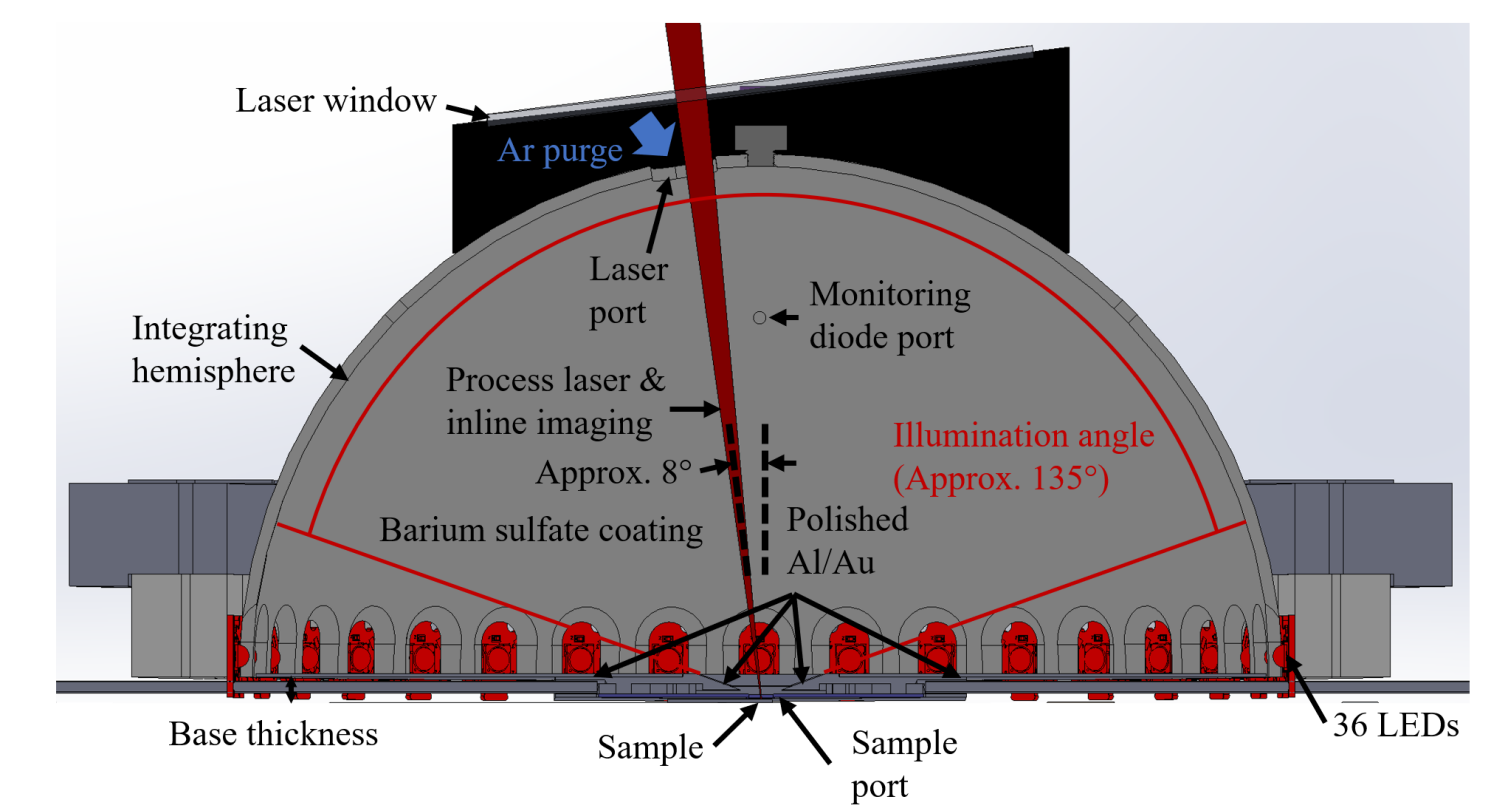		
**(b)**	**(c)**	**(d)**
** 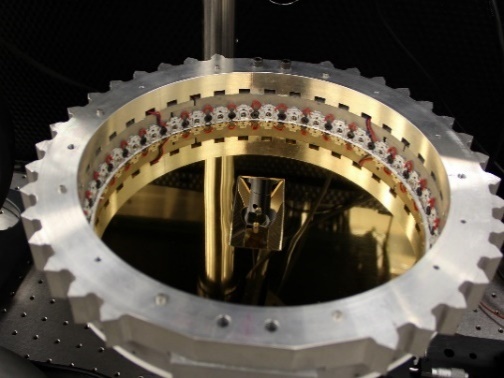 **	** 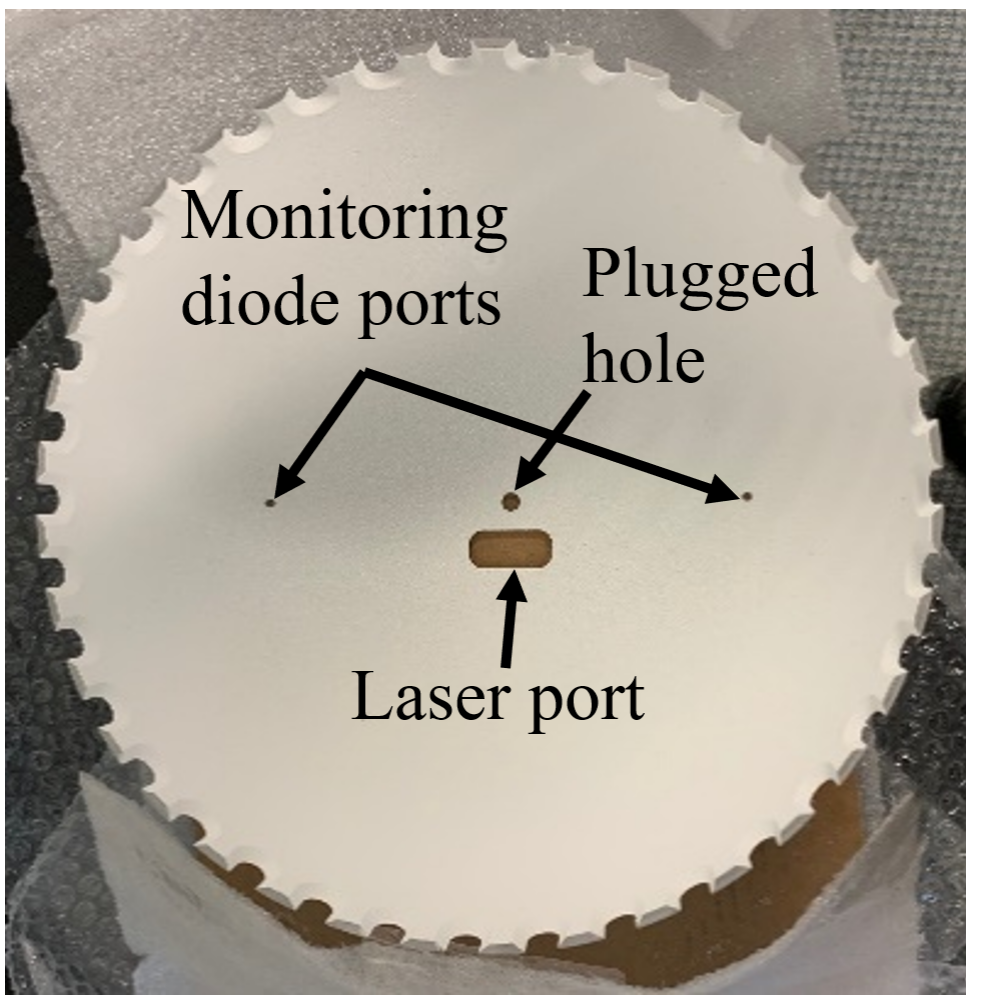 **	** 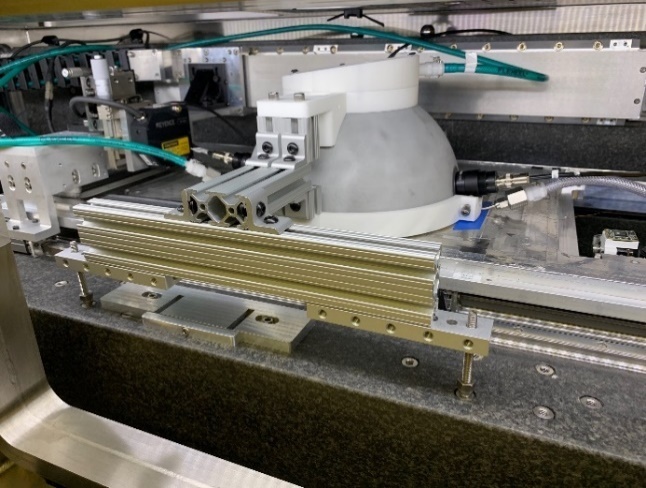 **
**Fig. 3.** (a) Cross-section view of a computer-aided design model of the integrating hemisphere. (b–d) Images of the (b) specular base of the fabricated integrating hemisphere, (c) barium sulfate–coated hemisphere interior, and (d) assembled apparatus in build chamber.		

#### 3.3.1 Nonuniform and Incomplete Hemispherical Illumination

##### 3.3.1.1 Throughput Uniformity

Reflectometer throughput is the ratio of the flux reaching the detector to the input flux from the source. Relative throughput is measured across the integrating surface and reported in arbitrary units. Localized relative throughput mapping of the inside of the hemisphere quantifies the uniformity of hemispherical illumination of the reflectometer as a whole and allows estimation of the associated component of measurement uncertainty contributing to the overall emissivity measurement uncertainty.

Throughput mapping is performed with illumination around the equator of the hemisphere by 36 LEDs at 850 nm central wavelength. A photodetector is then placed at the entrance port, and a gimbal-mounted mirror is mounted at the sample port. The mirror is aimed at a representative number of locations across the internal surface of the hemisphere.

**Fig. 4:**
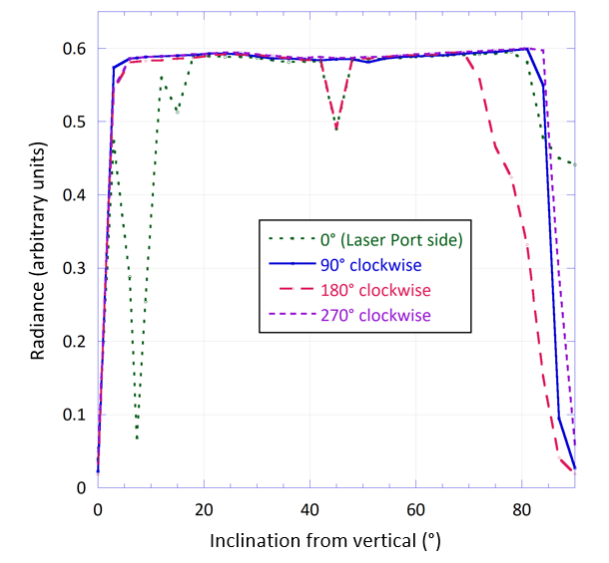
Relative throughput of the hemispherical integrating surface at four orthogonal directions. Angles of the sections are referenced to the polar angle around the equator of the hemisphere, and the inclination (azimuthal angle) is referenced to vertical in the reflectometer orientation shown in Fig. 3. The tests are performed with the specular gold base.

As shown in Fig. 4, the throughput uniformity of the surface is within 3.3% between approximately 5° and 80° from vertical. Decreased radiance throughput occurs at the laser port (5° to 15°) and at the monitoring diode ports (45°), and increased high-angle losses occur opposite to the port at 70° to 90°. The main features and nonidealities of the reflectometer are detected at these locations, and the uniformity around these features is assumed to be representative of the remainder of the reflective surface. Throughput uniformity is assumed to incur negligible bias in emissivity; therefore, the expected value is approximated by the center of the uncertainty distribution. The nominal value of $C_{TU}$ in Eq. (24) is assumed to be equal to 1, and its relative range is assumed to be ± 3.3% with uniform probability distribution, resulting in a relative standard uncertainty (normal distribution) of *u* = 0.033/√12 = 0.01, or $C_{TU}$ = 1 ± 0.01.

##### 3.3.1.2 Port Losses and High-Angle Losses

Incomplete hemispherical illumination is caused by loss of illumination light through the laser port and due to the base thickness, which causes lack of illumination at high angles. The change in reflection intensity due to incomplete illumination is measured by comparing the measured imager signal with a specular (silver) reflectance standard and a diffuse (gold) reflectance standard. A specular reflectance sample does not result in any port loss or high-angle loss, because it is illuminated only by a small portion of the illuminating surface of the reflectometer, opposite to the laser/imaging port. In contrast, a perfectly diffuse sample results in both high-angle and port losses, because it reflects illuminating light from the full hemisphere. After accounting for the reflectivity of the samples, it is found that the discrepancy is 2.5% in intensity that is lost from the specular sample to the diffuse sample. The laser-metal interaction scene is likely better represented by a value between specular and diffuse, so the bias is assumed to be halfway between the two samples. Plus or minus half of the difference is assumed to represent the standard uncertainty with a normal distribution. Therefore, the nominal value of $C_{PL}$ is 1.013 with a standard uncertainty of 0.013.

The reflective character of the laser-metal interaction scene is a significant unknown and could potentially result in more port and high-angle losses under certain laser process conditions than are estimated here. Therefore, two approaches have been identified to better quantify the emissivity uncertainty component due to port losses, and one approach has been identified for estimating high-angle losses.

The first approach to estimating port losses is to reduce the size of the laser port to as small as possible, which would result in a reduced scan distance. The relative change in signal compared with the normal port size can then be used to calculate a more representative port loss. The second approach is to add a supplementary light source *via* a beam splitter directed along the imaging/laser path to add additional light from the port area, which will then be optically integrated by the reflectometer. The additional signal may then be used to better quantify port loss bias and uncertainty.

To estimate high-angle losses, a small sample is moved upward through the base of the reflectometer with an adjustable cylindrical baffle surrounding it. The motion of the sample relative to the baffle and reflectometer base and the resulting change in signal intensity due to the change in illumination from the LEDs set at constant power are used to quantify the effect of high-angle losses. The high-angle losses are expected to be negligible under most circumstances, but the high-angle loss test raises concerns about scattering and absorption in the plume generated above the melt pool during laser scan tests. Questions arise regarding how to deconvolve those effects from high-angle loss effects, which must be addressed in future work. Nevertheless, the combined port loss and high-angle loss estimate reported here is believed to be acceptable under most conditions.

##### 3.3.1.3 Coating Reflectance and Diffuseness

The reflectance and specularity of the integrating sphere coating can generate measurement uncertainty, with more reflective and more diffuse coatings generating more accurate measurements [25, 26]. The barium sulfate coating used in this application has highly diffuse reflectance with hemispherical reflectance of 0.981 at 850 nm wavelength [27]. Therefore, the coating reflectance and diffuseness are expected to have a negligible effect on the accuracy of measurements in the given application, and they are therefore omitted in Eq. (24) and the uncertainty budget.

##### 3.3.1.4 LED Reproducibility

The intensity and spectrum of the hemispherical illumination LEDs change as the junction temperatures increase within the semiconductor devices. According to the manufacturer datasheet, the radiant flux output decreases by as much as 35% when the LED case temperature varies from room temperature to 100 °C, and the peak wavelength shifts by 13 nm [28]. The absolute shifts in output and wavelength are not of high importance alone, but the reproducibility of the output from the measurement of the reflectance standard to the measurement of the sample introduces an uncertainty to the emissivity measurement. The reproducibility of the LED illumination was tested previously with a cursory series of repeat tests of LED illumination, from which the estimated standard uncertainty in the LED correction factor $C_{LED}$ was 1.5%. Stochastic non-reproducibility does not induce bias, and, therefore, the nominal value of $C_{LED}$ is 1.0 with standard uncertainty of 0.015.

The type A uncertainty component associated with LED reproducibility can be measured with statistically significant repeat tests using the reflectance standard while recording the signal received by the imager. This uncertainty component can also be reduced by combined triggering of the LEDs with triggering of the imager and laser scanning system.

#### 3.3.2 Reference Mirror Alignment and Reflective Character

##### 3.3.2.1 Reference Mirror Gap and Alignment

**Fig. 5:**
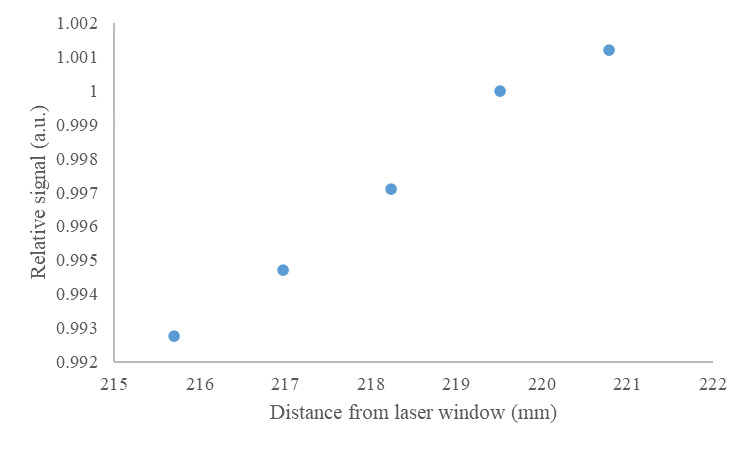
Relative signal measured as a function of specular reflectance standard location relative to the laser window, with the floor of the reflectometer located at 219.3 mm.

As discussed previously, both specular and diffuse samples are used in the emissivity measurement and evaluation of uncertainties. The angular alignment of the reference mirror surface and its distance from the specular base through the reflectometer sample port (gap) slightly alter the illumination of the reflector. In the case of the specular mirror, misalignment or altered gap change the location on the integrating surface from which the sample is illuminated, while also reducing throughput due to light loss from the gap. An experiment was performed to measure the relative change in signal when the specular reflectance standard is moved relative to the floor of the reflectometer (located at 219.3 mm from the laser window), as shown in Fig. 5.

In the device setup, the reference mirrors are aligned with a high-resolution (0.1 µm) laser displacement meter (vendor-stated accuracy ±0.5 μm), and the reflectometer is mounted on kinematic mounts. Rotational misalignment is believed to be negligible. It is conservatively estimated that the reference mirror is manually located within ±1 mm; the relative range of signal is 0.6%. The signal has a uniform probability of being within that range, so dividing by $\sqrt{12}$, the signal uncertainty is 0.2% [29]. Therefore, the nominal value of $C_{ARM}$ is 1.0 with standard uncertainty of 0.002.

##### 3.3.2.2 Reference Mirror Reflectance

The reflectance standards used in these experiments are calibrated by NIST to have a nominal reflectance $\rho_{ref}$ of 0.97. The standard uncertainty of reflectance value is reported as 0.5% (or 0.005) [30].

#### 3.3.3 Sample Alignment and Reflective Character

##### 3.3.3.1 Sample Gap and Alignment

**Fig. 6:**
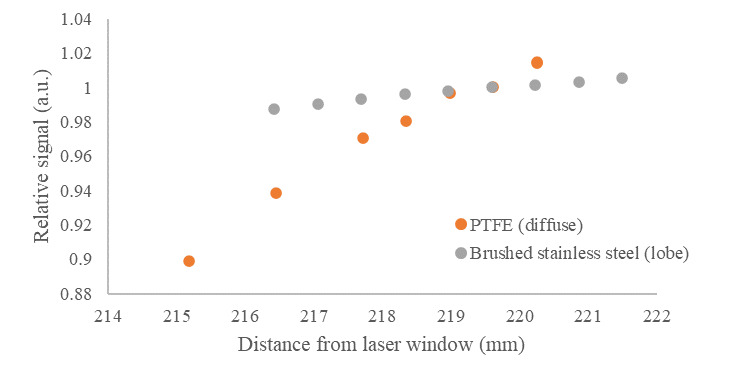
Relative signal measured as a function of the location of two reflectance standards relative to the laser window, with the floor of the reflectometer located at 219.3 mm.

The effects of sample gap changes and misalignment are similar to those of the reference mirror, in that this affects the area on the dome surface from which the sample is illuminated, which results in slight changes in reflection intensity. Currently, the best approximation of the effect of sample alignment is shown by measurements taken with a diffuse reflective sample (pressed polytetrafluoroethylene [PTFE]) and a sample with a lobed reflective character between that of specular and diffuse (brushed stainless steel). The results of gap change between the reflectance standards and the laser window (with the reflectometer floor located at 219.3 mm from the laser window) are shown in Fig. 6.

The samples are aligned with the high-accuracy and high-resolution (0.5 µm and 0.1 μm, respectively) laser displacement sensor to an expected precision of ±20 µm, and, again, the reflectometer is mounted on kinematic mounts. Rotational misalignment is believed to be negligible. If it is conservatively estimated that the specular reflectance standard is located within ±1 mm, and the diffuse reflectance standard is representative of the maximum change associated with a real sample’s change in reflectance (*e.g.*, stainless steel reflectance sample or a melt pool), the relative range of signal is approximately 5.0%. Assuming a uniform probability distribution within this range, the standard uncertainty of $C_{AS}$ becomes 1.5%. Therefore, $C_{AS}$ = 1.0 ± 0.015.

##### 3.3.3.2 Sample Bidirectional Reflectance Distribution Function

The sample BRDF is a mathematical function that describes how the hemispherical illumination of the sample is reflected and imaged by the directional imaging system. Currently, very little is known or has been measured regarding the reflective character of the laser-metal interaction scene, and this is an important area for future investigation. In the absence of additional information, the sample BRDF is assumed to have a similar effect to the throughput uniformity with no bias. Therefore, $C_{BRDFS}$ = 1.0 ± 0.01. It should be noted that in the unlikely circumstance that the majority of light reflected from the sample originates from very high angles or from the imaging port, the sample BRDF could be a dominant uncertainty component.

##### 3.3.3.3 Sample Substitution Error

Sample substitution error occurs due to the difference in reflectance between the reference mirror and sample. When the reference mirror is replaced with the lower reflectance sample, the total throughput of the reflectometer decreases, which reduces the measured reflectance of the sample (increasing the measured emissivity). The bias in measured reflectance was calculated with a method similar to that given by Vidovic and Majaron [31]. The substitution error was calculated to be approximately 0.5%. Therefore, $C_{Sub}$ = 1.0025 ± 0.005.

#### 3.3.4 Directional Imaging

##### 3.3.4.1 Out-of-Field Scatter from Sample Port Surfaces

The edges of the reflectometer sample port are a nonideality that may cause out-of-field scatter that may be detected as erroneous signal by the directional imaging system. This effect is expected to be small, and in the absence of experimental data, it is assumed to induce an increase of signal bias of 0.25% with a standard uncertainty of 0.5%. Therefore, $C_{S}$ = 0.9975 ± 0.005.

##### 3.3.4.2 Polarization Effects

Polarization may significantly alter the throughput uniformity of integrating spheres and should be considered as a potential uncertainty component [32]. The integrating hemisphere uses 36 symmetrically distributed LED sources for illumination, which means that the illumination is very unlikely to have a preferred polarization illumination of the sample or reference mirror. The samples used for melt pool generation are randomly sanded, making the unmelted material unlikely to have preferred polarization. The silver reference mirror has very low polarization at 850 nm wavelength and at the reflection angle of 8° from normal, making reference mirror polarization bias unlikely. Finally, nearly all practically useful data are obtained on molten or resolidified sample surfaces, which have potential for polarizing effects, but this has not yet been measured. In the future, series of tests will be performed with varying scan direction and rotation of the randomly sanded sample to quantify polarization effects. Currently, the measurement uncertainty of emissivity due to polarization is assumed to be negligible.

##### 3.3.4.3 Imager Signal Linearization and Uncertainty

The primary instrument used for temperature measurements is a Photron FastCAM Mini AX200^1^1 Certain commercial entities, equipment, or materials may be identified in this document to describe an experimental procedure or concept adequately. Such identification does not imply recommendation or endorsement by the National Institute of Standards and Technology, nor does it imply that the entities, materials, or equipment are necessarily the best available for the purpose. imager. The imager has a 1024 pixel × 1024 pixel array with a 12 bit dynamic range FPA. In this work, all data were obtained with a shutter speed (SS) of 98.3 µs and a spatial resolution of 6.0 µm per pixel. The transient pixel noise and nonuniformity across the FPA are sources of signal uncertainty. Furthermore, the nonlinearity of signal with incident flux requires correction by calibration to a known-flux source, which introduces an additional uncertainty in the signal, each of which will be discussed in this section (Sec. 3.3.4.3). It should be noted that any references to signal within this paper refer to linearized signal, except for this section (Sec. 3.3.4.3).

##### 3.3.4.4 Transient Pixel Noise

In order to evaluate the uncertainty caused by transient pixel noise, images from the high-temperature blackbody (HTBB) calibration are used. The imager is outfitted with an Infinity K2 Distamax long-working-distance microscope lens body with a CF1 objective lens focused at the HTBB aperture to generate uniform spatial and temporal irradiance on the FPA, so signal variation is primarily due to electronic noise. The spatial nonuniformity of blackbody irradiation is considered to be negligible in this evaluation. The GainCal function in the imager software is used for flat-field correction (*i.e.*, nonuniformity correction [NUC]) to reduce natural optical vignetting and improve pixel uniformity across the FPA.

A set of 100 images is taken with varying HTBB set-point temperatures, and then the standard error (SE) of the mean digital level (DL) of each pixel, ${SE}_{P}\left(i,j\right)$, is taken for the 100 image sample set. In this example, a detector field of view (FOV) of 412 pixels × 412 pixels is used, though this example applies to FOVs frames of varying size.

**Table:** 

	${SE}_{P}\left(i,j\right)=\frac{1}{\sqrt{99}}\sqrt{\frac{1}{100}\sum_{k=1}^{100}{\left(S\left(i,j,k\right)-\mu_{P}(i,j)\right)}^{2}}$	(26)

where the indices $\left(i,j\right)$ represent the pixel columns and rows in each frame, and the index *k* represents a frame number. The pixel mean ($\mu_{P}$) is given as:

**Table:** 

	$\mu_{P}(i,j)$ =$\;\frac{1}{100}\sum_{k=1}^{100}S\left(i,j,k\right)$	(27)

The SE of each pixel of one set of frames is shown in Fig. 7a. This was taken at an HTBB temperature resulting in an average signal level of 1 426 DL. The SE for each pixel is then averaged across the frame to evaluate the typical transient pixel noise at each average signal value, as shown by Eq. (28).

**Table:** 

	$\mu_{F}\left({SE}_{P}\right)=\sum_{j=1}^{412}\sum_{i=1}^{412}\frac{{SE}_{P}\left(i,j\right)}{412^{2}}$	(28)

This is calculated at each frame set taken at each calibration point (setpoint) on the HTBB. Results are shown with SE as a percent of the mean signal in Fig. 7b. Use of the dynamic range is limited to the range between 200 DL and 3 800 DL, which results in a transient pixel noise component of relative standard uncertainty in terms of signal equal to 0.3%.

**Fig. 7:** (a) SE of each pixel of typical calibration set with SS = 98.3 µs and (b) frame average transient pixel SE throughout the FPA dynamic range. **Table:** 

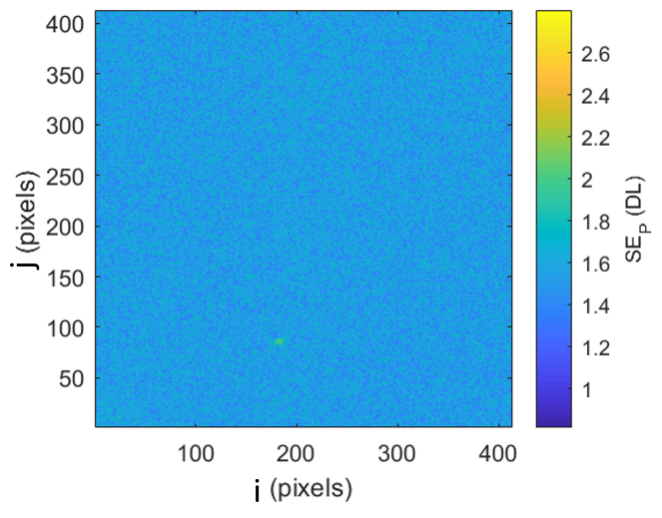 (a)	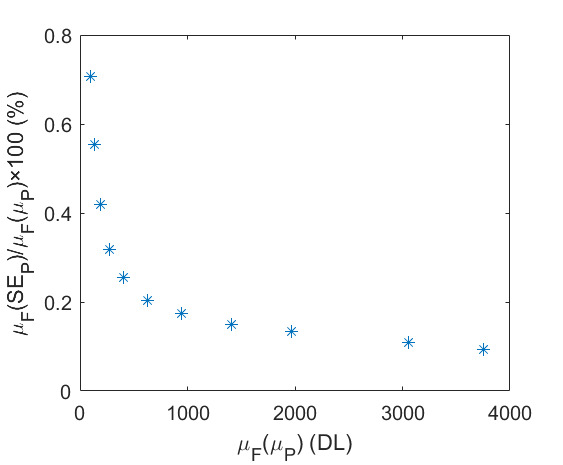 (b)

##### 3.3.4.5 FPA Nonuniformity

Images from calibration against the HTBB are used to characterize FPA nonuniformity. The pixel average is taken from a 100 frame set, resulting in a calibration image with the average DL of each pixel as shown in Fig. 8a. The standard deviation across the pixel array is then taken to evaluate nonuniformity across the FPA, as shown in Eq. (29).

**Table:** 

	$\sigma_{F}\left(\mu_{P}\right)=\sqrt{\sum_{j=1}^{412}\sum_{i=1}^{412}\frac{{\left(\mu_{P}\left(i,j,k\right)-\mu_{F}\left(\mu_{P}\left(i,j\right)\right)\right)}^{2}}{412^{2}}}$	(29)

The resulting frame standard deviation of pixel average is expressed as a percentage of DL in Fig. 8b. Use of the dynamic range is limited to the range between 300 DL and 3 800 DL, which results in an FPA nonuniformity component of standard uncertainty of 1.0%. The standard signal uncertainty due to transient pixel noise and signal standard uncertainty due to FPA nonuniformity are then combined using the root-sum-of-squares (RSS) method, resulting in a combined standard uncertainty of 1.05%.

**Fig. 8:** (a) Typical pixel average image from 100 frames with SS = 98.3 µs and (b) frame standard deviation of pixel average across the FPA and throughout the dynamic range. **Table:** 

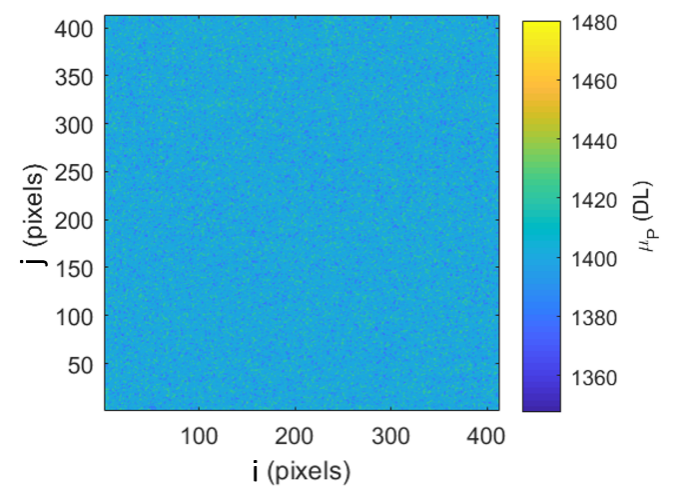 (a)	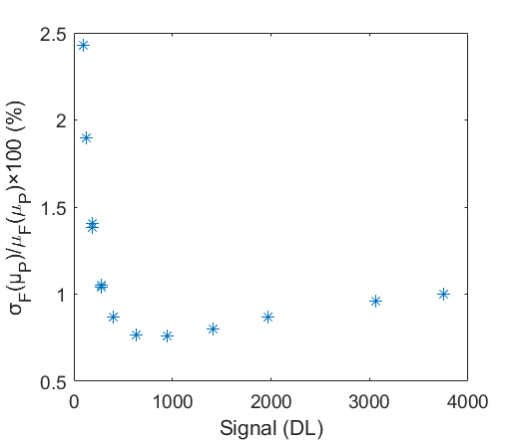 (b)

##### 3.3.4.6 Linearization with Radiant Flux

As discussed previously, the imager signal must be linearized to be proportional to the incident flux for accurate measurement of object radiance from which temperature is calculated. The measurement equation for camera calibration to the blackbody with an Infinity K2 lens body with CF1 objective lens, band-pass filter, and laser cutoff filter is as follows:

**Table:** 

	$S_{cal}\left(T\right)=C_{cal,BB}(T)\int_{\lambda 1}^{\lambda 2}\tau_{\lambda }^{CF1}\tau_{\lambda }^{filt}r_{\lambda }^{FPA}\;Pl\left(\lambda ,T_{rad,BB}\right)d\lambda$	(30)

Here, $T_{rad,BB}\;$is the known radiant temperature of the HTBB, based on a traceably calibrated correction factor relating $T_{rad,BB}$ to the HTBB setpoint temperature. Signal values during calibration are evaluated using the frame average of the pixel average, resulting in a single signal value from 100 images at each HTBB temperature. This approach is appropriate because of the relatively low noise and FPA nonuniformity discussed in the previous two subsections of Sec. 3.3.4.3. The single value is determined by the frame average of the pixel average as follows:

**Table:** 

	$S_{cal}=\mu_{F}(\mu_{P})=\sum_{j=1}^{412}\sum_{i=1}^{412}\frac{\mu_{P}(i,j)}{412^{2}}$	(31)

For linearization, the blackbody calibration constant $C_{cal,BB}$ must be established. Equation (30) is then rearranged as follows:

**Table:** 

	$C_{cal,BB}(T)=\frac{S_{cal}(T)}{\int_{\lambda 1}^{\lambda 2}\tau_{\lambda }^{CF1}\tau_{\lambda }^{filt}r_{\lambda }^{FPA}\;Pl\left(\lambda ,T_{rad,BB}\right)d\lambda }$	(32)

In order to solve Eq. (32), the lens spectral transmittance ($\tau_{\lambda }^{CF1}$), filter spectral transmittance ($\tau_{\lambda }^{filt}\left(\lambda \right)$), and FPA spectral responsivity ($r_{\lambda }^{FPA}$) must be measured. Inspection of Eq. (32) shows that uniformly scaling these spectral quantities across the spectral band of interest (*λ1→λ2*) will change the calibration constant ($C_{cal,BB}(T)$) proportionally. Therefore, the spectral functions can be measured in arbitrary units without consequence on the accuracy of calibration of the signal ($S_{cal}(T)$) to the HTBB temperature ($T_{rad,BB}$). The same concept applies to bias of measurement of the spectral functions within the uncertainty of the measurement—uniform bias across the spectral band (*λ1→λ2*) does not contribute to calibration uncertainty. Conversely, a significant potential for calibration uncertainty exists if the measured spectral functions vary from the nominal values minus the measurement uncertainty to the nominal values plus the measurement uncertainty (or vice versa) within the spectral band of interest. Evaluation of the calibration uncertainty due to this effect of measurement uncertainty in the spectral functions is described next.

The measured relative spectral responsivity of the FPA, $r_{\lambda }^{FPA}$, is shown in Fig. 9a. The instrument used to measure the responsivity is conservatively estimated to have standard uncertainty of 2.0%. The worst cases of the value varying from its minimum value of 0.98$r_{\lambda }^{FPA}$ to 1.02$r_{\lambda }^{FPA}$, corresponding to the wavelength range 830 nm to 870 nm, is also shown in Fig. 9a. Because of the wide and uneven spectral spacing of the data points, interpolation of the values is also shown. All values of the spectral quantities used in Eq. (32) are evaluated at all wavelengths within the spectral band with 1 nm increments, and their product is integrated using trapezoidal summation.

The measured spectral transmittance of the combined FBH850-40 band-pass filter and FES1000 laser cutoff filter is shown in Fig. 9b. The spectrometer used to measure the transmittance is known from experience to have a standard uncertainty of 0.5%. The worst cases of the value varying from its minimum value of 0.995$\tau_{\lambda }^{filt}$ to 1.005$\tau_{\lambda }^{filt}$ from 830 nm to 870 nm are also shown in Fig. 9b. Finally, the relative transmittance of the CF1 lens assembly ($\tau_{\lambda }^{CF1}$) is estimated to have conservatively large standard uncertainty of 2.0%, as shown in Fig. 9c.

The uncorrected signal data as a function of HTBB temperature are shown in Fig. 10a. The calibration constant $C_{cal,BB}$ is evaluated at the calibration point with the smallest uncertainty, which is 632 DL, in Eq. (32). Equation (30) is then evaluated with $C_{cal,BB}$ at each HTBB temperature, resulting in the linearized signal values shown in Fig. 10a. The correction function to convert uncorrected signal into linearized signal ($C_{lin}\left(S(T)\right)$) is shown by Eq. (33) and plotted in Fig. 10b.

**Fig. 9:** (a) Measured relative spectral responsivity of the FPA ($r_{\lambda }^{FPA}$), (b) measured relative spectral transmittance of the combined FBH850-40 band-pass filter and FES1000 laser cutoff filter, and (c) the assumed relative spectral transmittance of the CF1 lens assembly. Measured quantities include linear interpolation between measurement points. Worst case increasing/decreasing refers to linear variation of the relative spectral quantities from minimum to maximum uncertainty across the spectral band of interest for uncertainty analysis. **Table:** 

	$C_{lin}\left(S(T)\right)=\;S_{lin}\left(T\right)/S(T)$		(33)
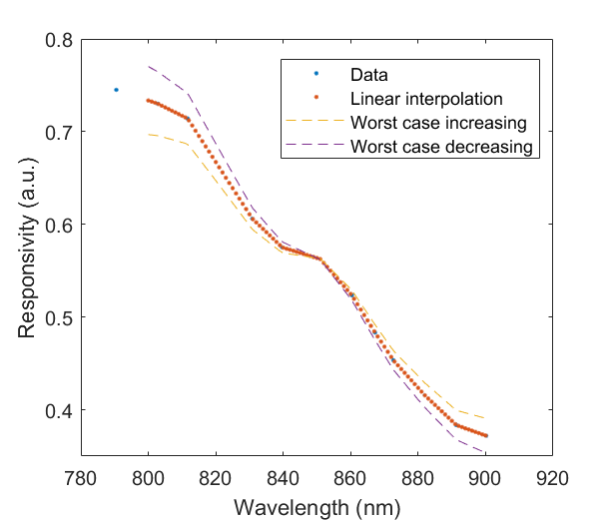 (a)		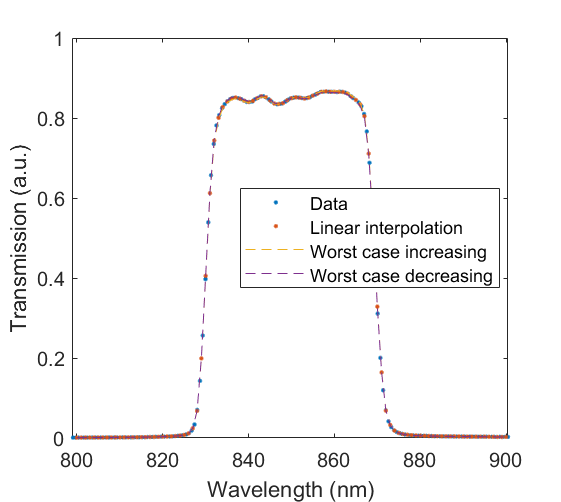 (b)	
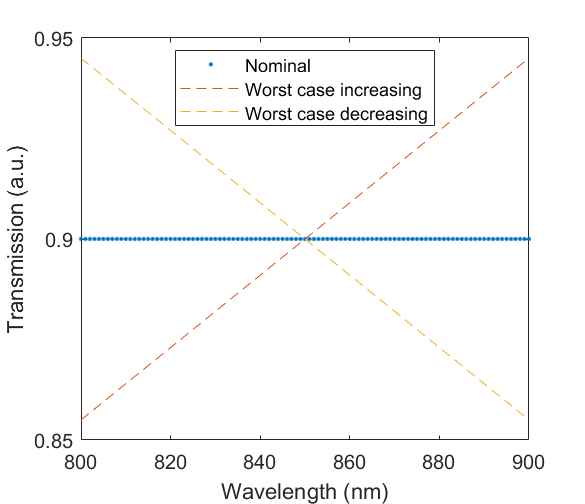 (c)

##### 3.3.4.7 Combined Signal and Linearity Uncertainty

The uncertainties of$\;\tau_{\lambda }^{filt}\left(\lambda \right)$, $\tau_{\lambda }^{CF1}\left(\lambda \right)$, $r_{\lambda }^{FPA}\left(\lambda \right)$, and ${S\left(T\right)}_{cal}$ are quantified as described in the preceding sections. The uncertainty of the HTBB radiance ($L_{\lambda }^{BB}\left(\lambda \right)$) will be described in detail in Sec. 4. The uncertainty of the wavelength (λ) is assumed to be equivalent to the resolution of the spectrometer.

**Fig. 10:** (a) Linearized (corrected) and uncorrected FPA signal as a function of HTBB temperature and (b) signal correction factor ($C_{lin}\left(S(T)\right)$ as a function of uncorrected signal value. **Table:** 

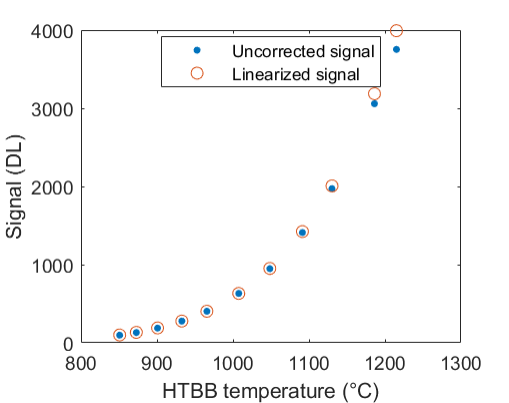 (a)	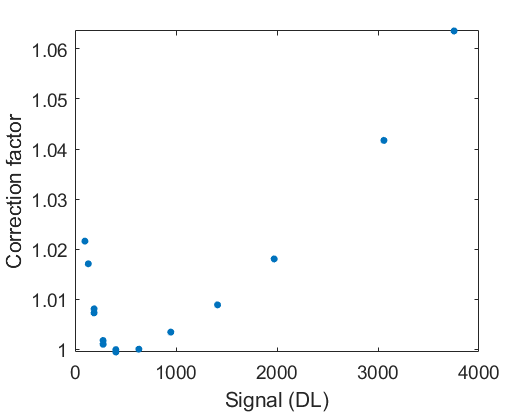 (b)

The combined uncertainty of the calibration constant is calculated by evaluating Eq. (32) with each variable in its worst case. This establishes the sensitivity of the calibration constant to each uncertainty component. Then, the sum-square of all of the uncertainty components is used to evaluate the combined uncertainty of the calibration constant. The uncertainties are treated as uncorrelated. These results are shown in Table 1.

**Table 1:** Signal and linearity uncertainty component effects on the calibration constant.

Variable	Uncertainty (*k =* 1)	Type	Change from Nominal Value	Change in $C_{cal,BB}$ (%)
$\tau_{\lambda }^{filt}\left(\lambda \right)$	0.5%	B	Increasing with λ	−0.01
			Decreasing with λ	0.01
$\tau_{\lambda }^{CF1}\left(\lambda \right)$	2.0%	B	Increasing with λ	−0.05
			Decreasing with λ	0.05
$r_{\lambda }^{FPA}\left(\lambda \right)$	2.0%	B	Increasing with λ	−0.05
			Decreasing with λ	0.05
				
λ	0.5 nm	A	Absolute increase	−0.48
			Absolute decrease	0.48
$L_{\lambda }^{BB}\left(\lambda \right)$	0.6%	B	Absolute increase	−0.6
			Absolute decrease	0.6
${S\left(T\right)}_{cal}$	1.05%	A	Absolute increase	1.0
			Absolute decrease	−1.0
				
			Combined standard uncertainty of *C_cal,BB_*	1.25

Finally, the combined uncertainty of linearized signal can be evaluated from Eq. (33). The resulting standard uncertainty of linearized FPA signal, ${S\left(T\right)}_{lin}$, is 1.6%. The combined uncertainty of all uncertainty components of emissivity is discussed in Sec. 3.3.5.

#### 3.3.5 Combined Uncertainty of Emissivity at the Solidification Point of High-Purity Nickel

**Fig. 11:**
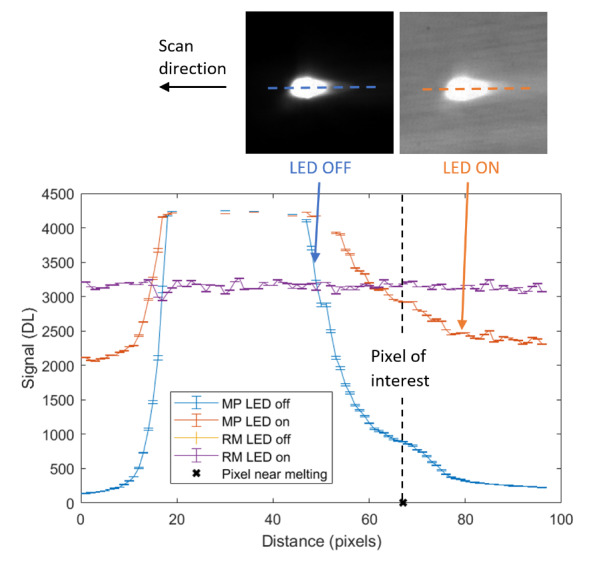
Image data of the melt pool (MP) generated with 99.998% Ni with LED on (top left), LED off (top right), and cross sections (lower middle). The DL values generated with the reference mirror (RM) with LED on and LED off at the same image coordinates are also shown, although the DL values with LED off are too low to be visible. The data parameters included laser power of 250 W, scan speed of 1 000 mm/s, D4σ spot size of 65 µm, image rate of 10 000 Hz, and 98.3 µs SS. Intensity profiles are average intensity of 30 images with SE of each pixel as error bars. The pixel to be evaluated is shown with a dashed line.

The measurement approach requires recording of image data in four steps. The melt pool is recorded with the inline imaging system with LEDs off, and then the procedure is repeated with LEDs on. Similarly, the reference mirror is imaged with LEDs off, and then the procedure is repeated with LEDs on. With melt pool tests, the frame rate (10 000 Hz here) and duration of laser scan (8 mm tracks at 1 000 mm/s scan speed here) produced image sets with 80 images or frames each. The melt pool requires some “development length” at the initiation of laser-melting, and it similarly has a cooldown time once laser power is turned off. Because of these considerations, the first 25 images and last 25 images are not used, and the central 30 images recorded during steady solidification are used. Image data of the melt pool generated with 99.998% Ni are shown in Fig. 11 with LED on (top right), LED off (top center), and intensity profiles along the centerline (middle). Intensity profiles are average intensity of 30 images with temporal SE of each pixel shown in the error bars. The pixel to be used in the single-pixel evaluation is shown with a vertical dashed line.

The current discussion is limited to evaluation of the uncertainty of one pixel. The cross section shown in Fig. 11 is used to identify a pixel as close to the solidification point, or inflection of the solidification plateau, as possible. Temperature and emissivity at the solidus point are of interest for determining cooling rates, and validation data are available for comparison at the known fixed-point solidification temperature of 99.998% Ni. Figure 11 also shows the signal generated with the reference mirror (RM) with LED on and LED off at the same image coordinates as the melt pool cross sections, although the signal generated with LED off is below the measurable range of the camera.

The combined uncertainty of the emissivity is calculated by first evaluating Eq. (24) with each variable in its worst case, or highest and lowest value resulting from measurement uncertainty. This establishes the sensitivity of emissivity to each uncertainty component. Then, the sum-square of all the uncertainty components is calculated to evaluate the combined uncertainty of the emissivity. The uncertainties are treated as uncorrelated. These results are shown in Table 2, with the conclusion that estimated uncertainty in emissivity is approximately 7.0% at the solidification point of nickel. It may be noted that the combined effects of the uncertainties are slightly nonlinear, but the asymmetry is small enough to be considered negligible. The uncertainty distribution is conservatively assumed to be Gaussian.

Note that the uncertainty of the signals includes the imager noise, nonuniformity, and linearization uncertainty, as well as process variability SE, but spatial effects on the uncertainty are not accounted for until Sec. 5. The data parameters included laser power of 250 W, scan speed of 1 000 mm/s, D4σ spot size of 65 µm, frame rate of 10 000 Hz, and 98.3 µs SS.

**Table 2:** Combined uncertainty of emissivity at solidification point of high-purity nickel.

Variable	Value	Units	Relative Uncertainty (*k =* 1) (%)	Type	Change in $\varepsilon_{\lambda }(\lambda_{o},T_{s})$ with High Value (%)	Change in $\varepsilon_{\lambda }(\lambda_{o},T_{s})$ with Low Value (%)
$C_{TU}$	1	—	1.0	A	−1.7	1.6
$C_{PL}$	1.013	—	1.3	B	−2.1	2.1
$C_{LED}$	1	—	1.5	B	−2.5	2.5
$C_{ARM}$	1	—	0.2	A	−0.4	0.4
$C_{AS}$	1	—	1.5	B	−2.5	2.5
$C_{BRDFS}$	1	—	1.0	B	−1.7	1.6
$C_{S}$	0.9975	—	0.5	B	−0.8	0.8
$C_{Sub}$	1.0025	—	0.5	B	−0.8	0.8
$\rho_{ref}$	0.97	—	0.5	A	−0.8	0.8
$S_{samp}^{LED\;On}\left(\lambda_{o},T_{s}\right)$	2912.8	DL	1.6	B	−3.9	3.9
$S_{samp}^{LED\;Off}\left(\lambda_{o},T_{s}\right)$	893.4	DL	1.8	B	1.4	−1.4
$S_{ref}^{LED\;on}\left(\lambda_{o}\right)$	3074.1	DL	1.6	B	2.6	−2.7
$S_{ref}^{LED\;off}\left(\lambda_{o}\right)$	0.1	DL	25.6	B	0.0	0.0
			Combined standard uncertainty		7.0	6.9

### 3.4 Validation and Discussion

This section (Sec. 3.4) describes the results of two experiments that were used to evaluate whether or not the measured emissivity values were within the standard uncertainty range that was established in the preceding subsections of Sec. 3. The first approach used non-melting samples and comparison of contact thermometry with two well-characterized samples. The second approach used measurement of emissivity of a laser-induced melt pool of high-purity Ni, which was then compared with published data.

#### 3.4.1 Indirect Validation of Emissivity by Comparison to Contact Thermometry

The first validation of the emissivity measurement is done by combining the emissivity and radiance measurements to extract the true temperature and comparing this value to a contact thermometry measurement. This approach, apparatus, and associated measurement uncertainties are based on those of Hanssen *et al.* [33]. A band-limited custom transfer source pyrometer (TSP850), described in detail in Sec. 4, is calibrated to the HTBB and used to measure the surface temperature of two samples. The solid samples in this case were SiC and oxidized nickel superalloy 600 that had a subsurface thermocouple temperature measurement. The samples were heated with a Na heat pipe-based heater.

At the temperatures of interest (approximately 900 °C), radiant heat losses are the dominant cause of a temperature gradient from the subsurface thermocouple to the surface that is measured by the radiometric approach. The surface temperature is corrected with the known physical properties of the materials by radiation and conduction heat transfer [33].

**Fig. 12:**
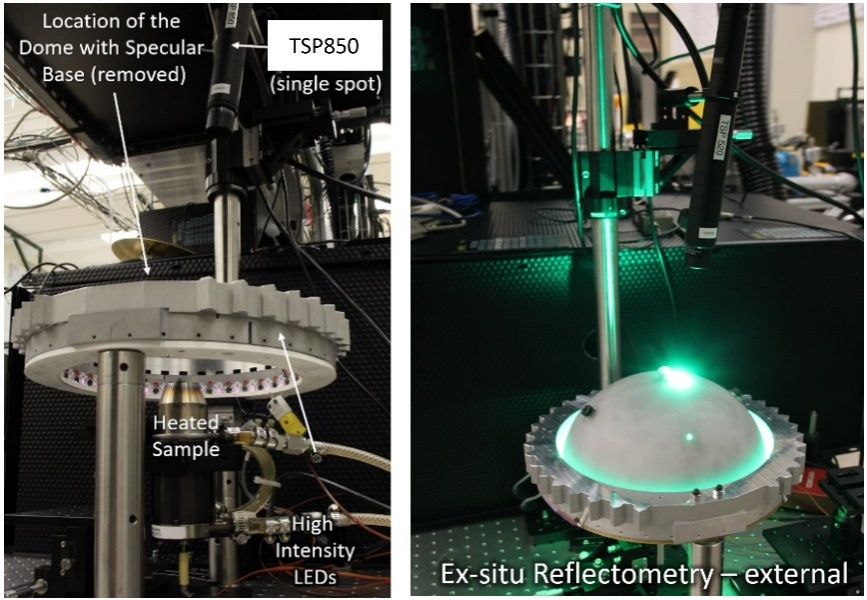
Experimental setup used to validate emissivity measurement using contact thermometry.

The emissivity of the sample surfaces is measured with the hemispherical reflectometer, and the radiance is measured with the TSP850, which is calibrated against the HTBB. The results are summarized in Table 3, and images of the experimental setup are shown in Fig. 12. The resulting difference is approximately 0.6 °C between contact thermometry and the radiometric-determined temperature of nearly 900 °C.

If the primary source of temperature measurement discrepancy were the emissivity measurement, the difference in emissivity value needed to correct the temperature error would be approximately 0.004 (emissivity is unitless as described in Sec. 1.1) . This emissivity difference corresponds to a reflectance (*ε = 1 − ρ*) error of 2% to 4%, which is in good agreement with the emissivity uncertainty established in Sec. 3.3.5.

Section 3.4.2 will compare measured emissivity at the solidification point of high-purity nickel as an additional validation of the emissivity measurement approach.

**Table 3:** Validation of emissivity measurement by comparison with contact thermometry.

Parameter	SiC Sample #1	Oxidized Nickel Superalloy 600
Radiance temperature measured with TSP850 (°C)	869.85	856.25
Measured spectral directional emissivity with integrating hemisphere	0.80	0.90
Measured surface temperature (°C)	884.91	867.23
Subsurface temperature measured by thermocouple (°C)	885.24	873.59
Thermal conductivity (W m^−1^ K^−1^)	134	28
Distance from subsurface thermocouple to surface (m)	0.0015	0.0025
Corrected surface temperature measured by subsurface thermocouple (°C)	884.28	866.65
Difference between radiometry and contact thermometry (°C)	0.63	0.58

#### 3.4.2 Comparison to the Emissivity of High-Purity Nickel at its Solidification Point

The measured value of emissivity near the solidification temperature (1 455 °C ± 1 °C) of 99.998% Ni generated by a scanned laser-induced melt pool is 0.376 with standard uncertainty of 0.026. Published data on similar material at 1 491 °C resulted in normal spectral emissivity of approximately 0.36 at 850 nm [34]. Therefore, under the conditions of comparison, the emissivity measurement approach developed here agrees with published values within the estimated measurement uncertainty. It should be noted that the measured emissivity and uncertainty reported in this section (Sec. 3.4.2) do not account for spatial effects on the measurement, which will be discussed in Sec. 5. Section 4 will describe temperature measurement of the same pixel.

## 4 Temperature Measured by a Pixel of the FPA

This section (Sec. 4) describes the calibration chain used to trace the temperature measurements conducted on the AMMT to the 1990 International Temperature Scale (ITS-90). The measurement equation will first be introduced, and then the uncertainty incurred at each step of the calibration methodology will be described. It should be noted that the measured temperature and uncertainty reported in Sec. 4 do not account for spatial effects on the measurement, which will be discussed in Sec. 5.

### 4.1 Measurement Equation

The measurement equation for an individual pixel viewing a uniform temperature field through the TEMPS optical train (although the approach discussed here is applicable to other optical systems) with an 850 nm band-pass filter (40 nm wide at half-height) and a laser cutoff filter is as follows:

**Table:** 

	$S_{samp}^{LED\;Off}(T_{samp})=C_{cal}\int_{\lambda 1}^{\lambda 2}\tau_{\lambda }^{TEMPS}r_{\lambda }^{FPA}\tau_{\lambda }^{filt}\varepsilon \left(\lambda \right)Pl\left(\lambda ,T_{samp}\right)d\lambda$	(34)

where the pixel signal$\;S_{samp}^{LED\;Off}(T_{samp})$ is the signal obtained without LED illumination (and is linearized as described in Sec. 3.3.4.3), $\tau_{\lambda }^{TEMPS}$ is the spectral transmittance of the TEMPS optics, $\tau_{\lambda }^{filt}$ is the filter spectral transmittance, and $r_{\lambda }^{FPA}$ is the imager spectral responsivity, $\varepsilon_{samp}\left(\lambda \right)$ is the spectral emissivity of the sample, and $Pl(\lambda ,T_{samp})$ is the calculated Planck function given in Eq. (7) and evaluated at the sample temperature $T_{samp}$. In order to solve Eq. (34) for temperature with discrete data, the integral is evaluated with trapezoidal summation on a uniform grid with 1 nm spacing, and the integral equation is iteratively solved with a gradient-based numerical solver. First, however, the calibration constant for the measurement equation, $C_{cal}$, must be determined. Section 4.1.1 will describe the calibration chain for the determination of $C_{cal}$.

#### 4.1.1 Calibrating the Imager and Optical Train

**Fig. 13:**
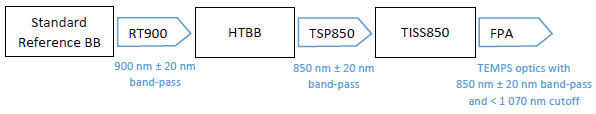
Calibration chain from the primary source to FPA calibration within the build chamber.

**Fig. 14:**
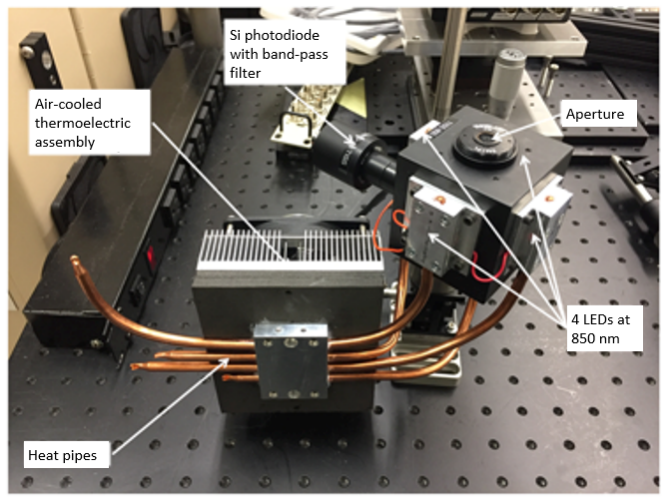
TISS850 transfer source for *in situ* calibration of FPA through TEMPS optics in the AMMT build chamber (shown outside of build chamber on an optics bench).

Calibration of the camera FPA in the TEMPS optical path is summarized in Fig. 13. The calibration is a multistage methodology that is referenced against primary standards at NIST. A NIST reference standard sodium heat-pipe blackbody (Na-HPBB) is used to compare a highly stable transfer pyrometer (RT900) to a reference thermometer that is calibrated against ITS-90 [14, 35]. The RT900 (which has a 900 nm central wavelength and 40 nm wide band-pass filter) is then used to calibrate the HTBB in the AMMT laboratory. The HTBB is then used to calibrate another transfer pyrometer (the TSP850, which has a 850 nm central wavelength and 40 nm wide band-pass filter), which is then used to calibrate a compact, portable transfer integrating sphere source (TISS850) that can be placed within the AMMT build chamber. The TISS850 (pictured in Fig. 14) is a transfer source developed in the AMMT that is composed of LEDs that are thermally stabilized (by ethanol heat pipes cooled by a thermoelectric cooler and fan) to illuminate a PTFE integrating sphere that generates uniform illumination at the waveband of interest with brightness that is calibrated against the HTBB thermal source. Finally, the TISS850 is used to calibrate the FPA through the TEMPS optics. The measurement equation of each calibration step is described in Sec. 4.1.2.

#### 4.1.2 Producing Calibration Coefficients

The measurement equation of the RT900 is as follows:

**Table:** 

	$S_{RT900}(T_{rad,NaBB})=C_{cal,RT900}\int_{\lambda 1}^{\;\lambda 2}r_{\lambda }^{RT900}Pl\left(\lambda ,T_{rad,NaBB}\right)\;d\lambda$	(35)

where $S_{RT900}$ is the photodetector signal, $r_{\lambda }^{RT900}$ is the combined relative spectral responsivity of the photodetector, optics, and filters of the RT900, and $Pl\left(\lambda ,T_{rad,NaBB}\right)$ is the calculated radiance at the Na-HPBB temperature $T_{rad,NaBB}$. Note that the integration limits, *λ1→λ2*, for the RT900 sufficiently encompass the 900 nm ± 20 nm responsivity. It may also be noted that the RT900 is calibrated in radiance temperature. Therefore, the emissivity of the Na-HPBB is not included in the measurement equation. The HTBB in the AMMT laboratory is then calibrated by the RT900 in radiance temperature ($T_{rad,HTBB}$), with the RT900 calibration constant established in the previous step ($C_{cal,RT900}$). The HTBB calibration equation is as follows:

**Table:** 

	$S_{RT900}(T_{rad,HTBB})=C_{cal,RT900}\int_{\lambda 1}^{\lambda 2}r_{\lambda }^{RT900}Pl\left(\lambda ,T_{rad,HTBB}\right)\;d\lambda$	(36)

Next, the TSP850 is calibrated against the HTBB, with a measurement equation as follows:

**Table:** 

	$S_{TSP}(T_{rad,HTBB})=C_{cal,TSP}\int_{\lambda 1}^{\lambda 2}\tau_{\lambda }^{FL}\tau_{\lambda }^{TSPfilt}r_{\lambda }^{PD}Pl(\lambda ,T_{rad,HTBB})d\lambda$	(37)

Note that in this case, the integration limits, *λ1→λ2*, for the TSP850 are 850 nm ± 50 nm in order to encompass the spectral range through which the band-pass filter transmits. $S_{TSP}$ is the photodetector signal, $\tau_{\lambda }^{TSPfilt}$ is the spectral transmittance of the 850 nm ± 20 nm band-pass filter used, $\tau_{\lambda }^{FL}$ is the spectral transmittance of the TSP850’s focusing lens, $r_{\lambda }^{PD}$ is the spectral responsivity of the TSP850’s photodetector, and $T_{rad,HTBB}$ is the radiance temperature of the HTBB. The calibration equation of the TISS850 band-limited source is then as follows:

**Table:** 

	$S_{TSP}(T_{rad,TISS})=C_{cal,TSP}\int_{\lambda 1}^{\lambda 2}\tau_{\lambda }^{FL}\tau_{\lambda }^{TSPfilt}r_{\lambda }^{PD}Pl(\lambda ,T_{rad,TISS})d\lambda$	(38)

where $T_{rad,TISS}$ is the radiance temperature equivalent to the brigthness generated by the TISS850’s LED-illuminated integrating sphere at the given waveband. The TISS850 is then placed *in situ* within the AMMT, and the FPA is set to image it through the TEMPS optics. The calibration equation of the camera FPA through the TEMPS optics is then:

**Table:** 

	$S_{cal}(T_{rad,TISS})=C_{cal}\int_{\lambda 1}^{\lambda 2}\tau_{\lambda }^{TEMPS}\tau_{\lambda }^{filt}r_{\lambda }^{FPA}Pl(\lambda ,T_{rad,TISS})d\lambda$	(39)

The camera calibration is performed using an integration time of 98.3 µs in this work. The uncertainty propagated through this calibration chain, and ultimately the uncertainty of the sample temperature measurement, will be discussed in the remainder of this section (Sec. 4).

### 4.2 Measurement Uncertainty

#### 4.2.1 RT900 Calibration

**Fig. 15:**
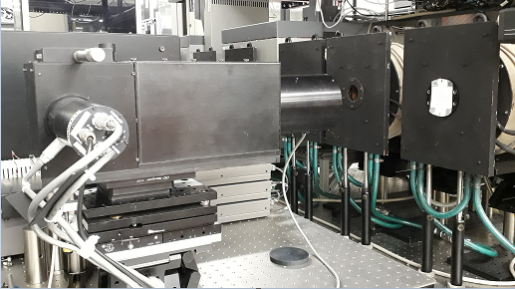
RT900 in position for calibration against the Na-HPBB.

The most recent calibration of the RT900 was performed with the NIST Na-HPBB at nominally 1 000 °C [14], as shown in Fig. 15. During calibration, the temperatures of the Na-HPBB are measured using two Au/Pt thermocouples, which are calibrated at NIST against the ITS-90 defined fixed points [35, 36]. The first thermometer is for temperature stabilization of the BB, and the second is used as an independent reference thermometer. In this way, the RT900 is calibrated against temperatures traceable to the ITS-90. The error due to the deviation in the Na-HPBB effective emissivity from the ideal value of unity is negligibly small when compared to the other measurement uncertainties. The Au/Pt thermocouples can be expected to measure values with a standard uncertainty of 0.047 °C [37]. During the calibration, the RT900 showed a maximum discrepancy of 0.02 °C compared with the reference thermocouples and had a measured temperature standard deviation of 0.03 °C over a 30 min period. These variations are taken as additional calibration uncertainties for a combined standard calibration uncertainty of 0.06 °C at a measurement temperature of 1 000 °C. In the initial validation of the RT900 at NIST in the early 2000s, the temperatures of primary standard Al and Ag fixed-point blackbodies (operating at 660.323 °C and 1 064.18 °C, respectively) were measured by RT900 to within 0.025 °C [38].

**Fig. 16:**
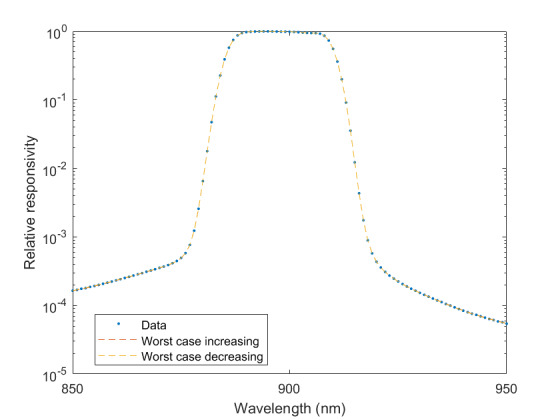
RT900 combined relative responsivity of the optics, filter, and detector.

In order to measure temperatures greater than 1 000 °C, the RT900 calibration must be extrapolated. The uncertainty in $C_{cal,RT900}$ due to the uncertainty of the temperature calibration is determined by evaluating Eq. (35) numerically at the nominal measured temperature plus or minus the measurement uncertainty. The uncertainty of $C_{cal,RT900}$ due to the maximum change in the relative responsivity across the 40 nm spectral band, estimated to be ±0.2%, is evaluated using the procedure described previously in Sec. 3.3.4.3. The relative responsivity of the RT900 combined optics, filter, and detector is shown in Fig. 16.

The uncertainties in the calibration constant are treated as uncorrelated and combined through the RSS method. The uncertainties of the RT900 calibration constant are summarized in Table 4, in which the combined standard uncertainty of $C_{cal,RT900}$ is found to be 0.061%.

**Table 4:** Combined uncertainty of the RT900 calibration constant.

Variable	Uncertainty (*k =* 1)	Type	Change in $C_{cal,RT900}$ with High Value (%)
$r_{\lambda }^{RT900}\left(\lambda \right)$	0.2%	A	0.016
$T_{rad,BB}$	0.06 °C	A	0.059
Combined standard uncertainty			0.061

Equation (35) is then used to determine the temperature uncertainty through calculation of the temperature range corresponding to the range of $C_{cal,RT900}$ values at ±0.061% of its nominal value. This analysis assumes that the RT900 pyrometer has a negligible nonlinearity in the radiance range of interest. The temperature uncertainty values of the RT900 at four values within the range of interest are shown in Table 5.

**Table 5:** RT900 temperature uncertainty (*k =* 1).

HTBB Temperature (°C)	Uncertainty (°C)
1 000	0.06
1 500	0.10
2 000	0.20
2 500	0.30

The uncertainty of the calibration of the HTBB by the RT900 is described in Sec. 4.2.2.

#### 4.2.2 HTBB Calibration

In addition to the calibration uncertainty of the RT900, several additional sources of uncertainty are encountered when the HTBB is calibrated against the RT900. The primary sources are the combination of radiance nonuniformity from the HTBB cavity and variation due to potential alignment error, the radiance/temperature variability within the cavity, the temporal noise estimated by the standard deviation of the pyrometer signal, and the spectral mismatch between the spectral band at which the RT900 is used in this work (40 nm width centered on 900 nm) and that of the HTBB (40 nm width centered on 850 nm).

**Fig. 17:**
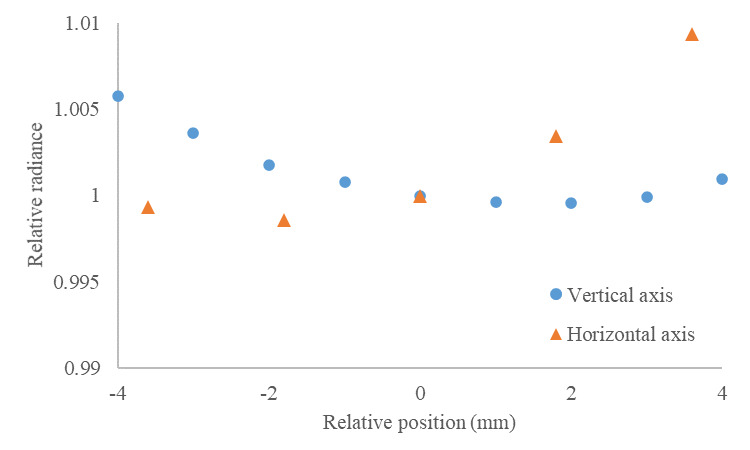
Relative radiance as a function of RT900 horizontal and vertical alignment to the HTBB aperture.

The effect of pyrometer alignment relative to the HTBB cavity is studied by varying the RT900 position relative to the HTBB aperture on translation stages, along both the horizontal and vertical axes. The relative radiance (radiance in arbitrary units) as a function of position is shown in Fig. 17.

Assuming a uniform probability of the RT900 being aligned within an 8 mm diameter area, the uncertainty component of the relative radiance with respect to position becomes 0.3%. This experiment is performed at nominally 1 200 °C and is assumed to be representative of the relative radiance nonuniformity from the HTBB cavity due to misalignment. The measured relative radiance is proportional to the calibration constant, so potential misalignment results in a relative standard uncertainty of 0.3% of the calibration constant, $C_{cal,HTBB}$.

The spectral radiance generated by the blackbody cavity has an uncertainty component due to temperature gradients that occur within the HTBB cavity. These stem from various heat losses/transfers, which depend on the HTBB operating temperature. For the HTBB used, the standard uncertainty in radiance due to temperature gradients is modeled and conservatively estimated to have a standard uncertainty of 0.5% [39].

The difference between the spectral radiances at 850 nm and 900 nm central wavelengths measured using 40 nm wide filters is another source of HTBB radiance uncertainty. The difference in the spectral radiance measured by the two pyrometers is primarily from the change in the spectral emissivity of the cavity material from one waveband to another. The emissivity of graphite is unlikely to introduce more than a 0.1% change in emissivity in this relatively narrow spectral mismatch [40]. This estimated standard uncertainty is incorporated in the uncertainty of $C_{cal,HTBB}$ by direct proportionality. Finally, the standard deviation in the pyrometer signal is typically 0.1% and is incorporated in the standard uncertainty of $C_{cal,HTBB}$ by direct proportionality. The uncertainties in the calibration constant are treated as uncorrelated and combined through the RSS method, with results shown in Table 6.

**Table 6:** Combined uncertainty of the HTBB calibration constant.

Uncertainty Source		Type	Uncertainty in$\;C_{cal,HTBB}$ (k = 1)
Nonuniformity/misalignment		A	0.3%
Cavity temperature gradients		B	0.5%
Signal/radiance standard deviation		A	0.1%
Spectral mismatch		B	0.1%
RT900 calibration		A	0.061%
	Combined standard uncertainty		0.60%

This uncertainty in the calibration constant, and correspondingly radiance, is converted into uncertainty in temperature for reference in Table 7.

**Table 7:** HTBB temperature uncertainty (*k =* 1).

HTBB Temperature (°C)	Uncertainty (°C)
1 000	0.6
1 500	1.2
2 000	1.9
2 500	2.9

#### 4.2.3 TSP850 Calibration

The TSP850 is calibrated against the HTBB, so the uncertainty of the radiance to which it is exposed is the combined uncertainty of the blackbody cavity radiance and the uncertainty stemming from misalignment. These additional relative standard uncertainties are 0.6% and 0.3%, respectively. The standard deviation of the signal is less than 0.01% of the signal and is an additional component of relative standard uncertainty.

The uncertainty of the TSP850 calibration constant, $C_{cal,TSP}$, due to spectral variability is analyzed here as was previously described in more detail in Sec. 3.3.4.3. The spectral responsivity of the TSP850’s photodetector (Hamamatsu S2281-04) is shown in Fig. 18a, along with estimated worst case variabilities in responsivity of 0.5% (*k* = 1) across the spectral band of interest [41]. The spectral transmittance variability of the focusing lens is conservatively estimated to be 2% (*k =* 1), as shown in Fig. 18b. Finally, the spectral transmittance of the FBH850-40 filter is shown in Fig. 18c, with an estimated standard uncertainty across the spectral band of 0.5%.

**Fig. 18:** (a) The manufacturer reported spectral responsivity of the TSP850 photodetector, (b) the assumed relative spectral transmittance of the TSP850 lens assembly, and (c) the manufacturer reported spectral transmittance of the combined TSP850 band-pass filter. The worst case increasing/decreasing curves show the linear variation of the relative spectral quantities from minimum to maximum uncertainty across the spectral band of interest for uncertainty analysis. **Table:** 

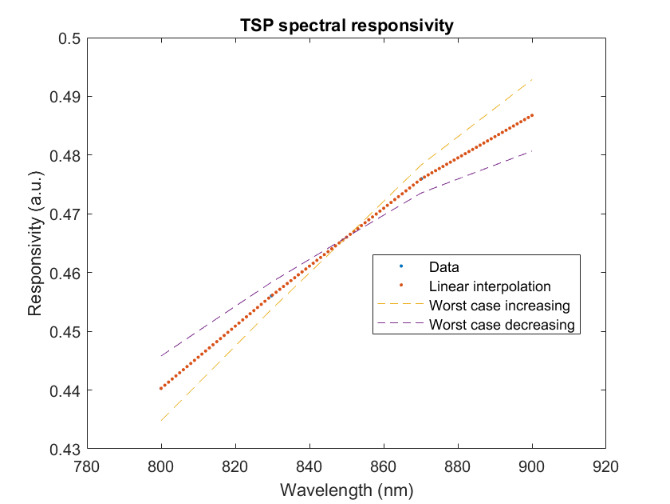 (a)	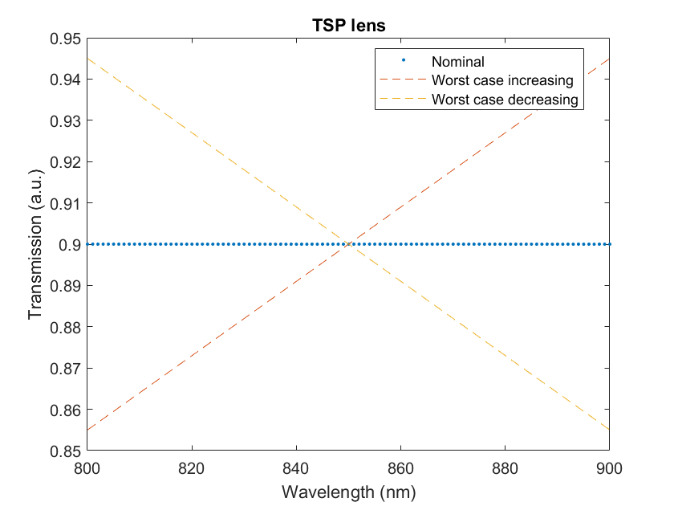 (b)
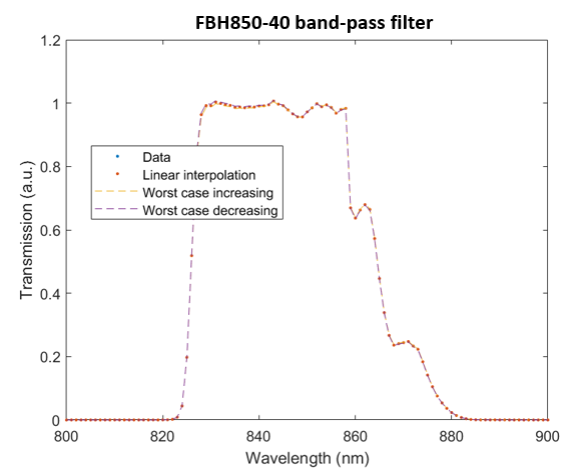 (c)

The uncorrected TSP850 signal data as a function of the HTBB temperature are shown in Fig. 19a. The calibration constant, $C_{cal,TSP}$, is evaluated at a central signal of 2 µA, given in Eq. (37). Equation (30) is then evaluated with $C_{cal,TSP}$ at each HTBB temperature, resulting in the linearized signal (proportional to incident radiant flux, not temperature) shown in Fig. 19a. The correction factor to convert the uncorrected signal into a linearized signal ($C_{lin,TSP}$) is shown by Eq. (40) and plotted in Fig. 19b.

**Fig. 19:** (a) Linearized (corrected) and uncorrected TSP850 signal as a function of HTBB temperature and (b) signal correction factor as a function of uncorrected signal value. **Table:** 

	$C_{lin,TSP}\left(S(T)\right)=\;S_{lin,TSP}\left(T\right)/S_{cal,TSP}\left(T\right)$		(40)
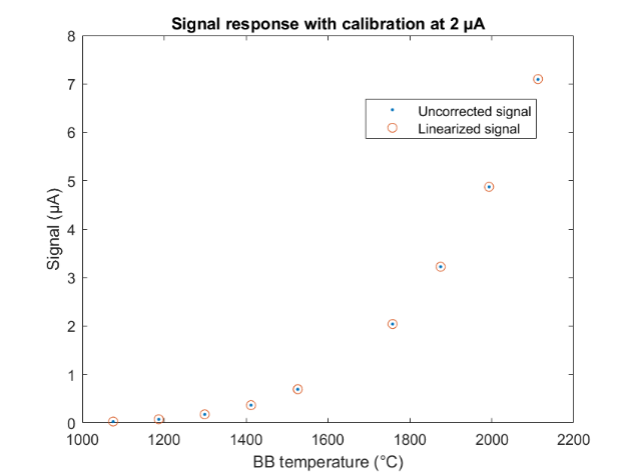 (a)		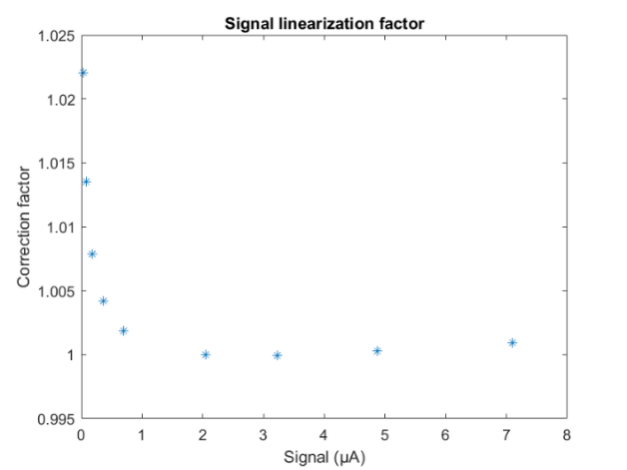 (b)

The signal variability of the TSP850 pyrometer calibrated against the HTBB is small, so the uncertainty contribution of noise to signals linearized by the correction factor ($C_{lin,TSP}$) is negligible. The results of the combined TSP850 calibration constant uncertainty are shown in Table 8.

**Table 8:** Combined uncertainty of the TSP850 calibration constant.

Uncertainty Source	Uncertainty (*k* = 1)	Type	Uncertainty in$\;C_{cal,TSP}$ (k = 1)
HTBB radiance	0.6%	B	0.6%
Misalignment	0.3%	A	0.3%
Signal/radiance standard deviation	0.01%	A	0.01%
Responsivity ($r_{\lambda }^{PD}\left(\lambda \right)$)	0.5% spectrally	B	0.08%
Lens transmittance ($\tau_{\lambda }^{FL}\left(\lambda \right)$)	2.0% spectrally	B	0.00%
Filter transmittance ($\tau_{\lambda }^{TSPfilt}\left(\lambda \right)$)	0.5% spectrally	B	0.08%
Wavelength (λ)	0.5 nm	B	0.2%
Combined standard uncertainty			0.7%

The uncertainty of transferring the TSP850 calibration to the TISS is described in Sec. 4.2.4.

#### 4.2.4 TISS850 Calibration

**Fig. 20:**
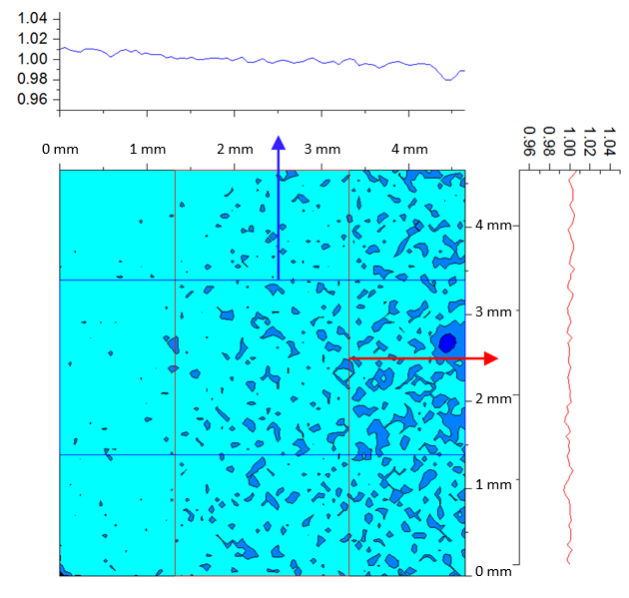
TISS850 uniformity within ±1 mm of the aperture center. Normalized representative cross sections are shown on the top and right.

The primary additional uncertainty component generated by calibration of the TISS850 by the TSP850 is due to the radiance nonuniformity of the TISS850 across its aperture, as shown in Fig. 20. This is measured by first exposing an FPA to the HTBB for field-flattening. The FPA is then exposed to the TISS within ±1 mm of the aperture center at a radiance temperature of 1 100 °C. The TISS850 nonuniformity at this radiance temperature is assumed to be representative of its nonuniformity throughout the temperature range of interest. Although there may be some systematic variability in the TISS850 uniformity, this nonuniformity is only a small component of the overall measurement uncertainty. So, although it may be possible to improve or adjust for TISS850 nonuniformity, the improvement in measurement accuracy would only be marginal. The average nonuniformity of the TISS850 radiance is found to be 0.1% and becomes an additional standard uncertainty component.

The same uncertainty components described in the calibration of the TSP850 against the HTBB occur in the calibration of the TISS850 against the TSP850: the signal/radiance standard deviation, the responsivity, the lens transmittance, the filter transmittance, and the wavelength uncertainties. The uncertainties in the calibration constant are treated as uncorrelated and combined through the RSS method. The components and combined standard uncertainty of the TISS850 calibration are summarized in Table 9.

**Table 9:** Combined uncertainty of the TISS850 calibration.

Uncertainty Source	Uncertainty (*k =* 1)	Type	Uncertainty in$\;C_{cal,TISS}$ (k = 1)
TSP850 radiance	0.7%	B	0.7%
TISS nonuniformity	0.1%	A	0.1%
Signal/radiance standard deviation	0.01%	A	0.01%
Responsivity ($r_{\lambda }^{PD}\left(\lambda \right)$)	0.5% spectrally	B	0.08%
Lens transmittance ($\tau_{\lambda }^{FL}\left(\lambda \right)$)	2.0% spectrally	B	0.00%
Filter transmittance ($\tau_{\lambda }^{TSPfilt}\left(\lambda \right)$)	0.5% spectrally	B	0.08%
Wavelength (λ)	0.5 nm	B	0.2%
	Combined standard uncertainty		0.75%

The uncertainty in calibrating the FPA with the TISS850 in the AMMT build chamber will be discussed next.

#### 4.2.5 FPA Calibration

As described previously in Sec. 3.3.4, the entire FPA is calibrated at once, and the spatial nonuniformity of the array and transient pixel noise are accounted for as standard uncertainties. All pixels are therefore treated identically, and so the following discussion addresses the calibration uncertainty of a single (typical) pixel.

It is found that when the FPA is exposed to the TISS850 through the TEMPS optics, the FPA noise and nonuniformity are comparable to the values produced when the FPA is exposed to the HTBB through the CF1 lens. The standard signal uncertainty due to noise and nonuniformity is 1.05%, and the standard uncertainty of the linearized signal is 1.6%.

**Fig. 21:**
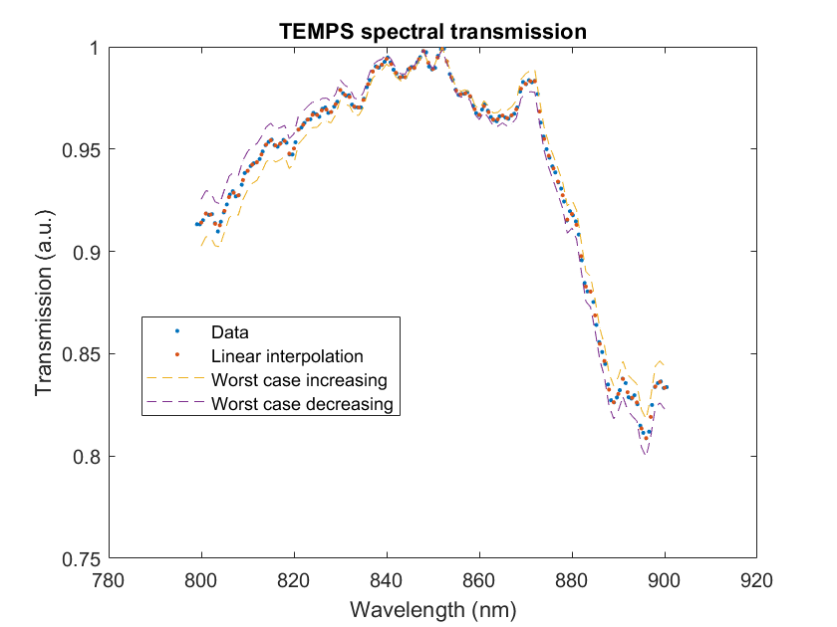
Transmittance of the TEMPS optics.

The uncertainties due to spectral effects in the filter transmittance, the FPA responsivity, and the inline optics are handled as described in Sec. 3.3.4.3. The spectral transmittance of the TEMPS optics is shown in Fig. 21, along with the worst cases of increasing and decreasing change in transmittance due to the spectrometer uncertainty across the spectral range of interest.

**Fig. 22:**
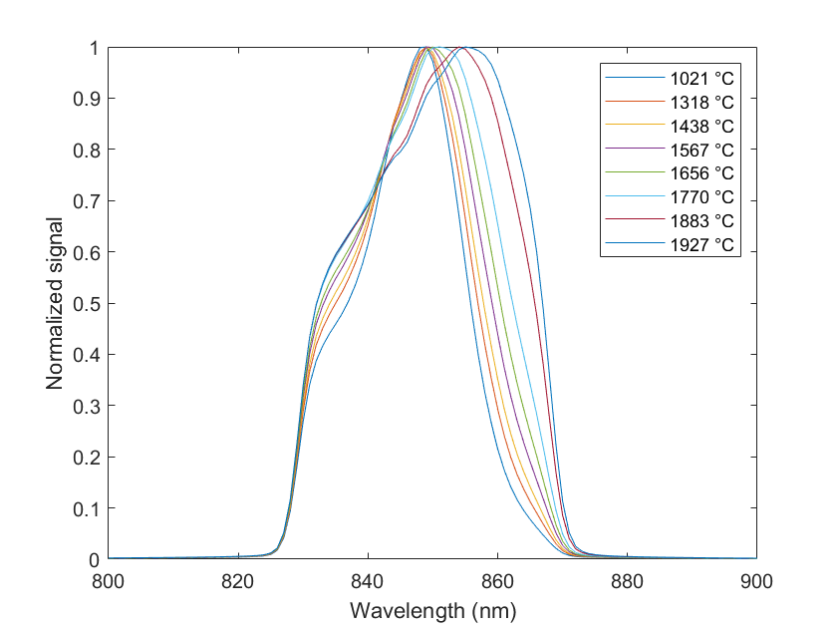
The TISS850 relative spectral radiance dependence on radiance temperature.

The selective emission of LEDs changes with increasing power and heat dissipation, resulting in an uncertainty in the FPA calibration unique to the TISS850. The normalized spectral emission of the TISS850 as a function of wavelength and radiance temperature generated at the aperture and through the standard 850 nm band-pass filter is shown in Fig. 22.

The FPA is calibrated against the TISS through the TEMPS optics to evaluate the effect of the changing spectral nature of the TISS850 radiance. Using Eq. (39), the calibration constant,$\;C_{cal}$, is calculated from the linearized average FPA signal at each TISS850 temperature. The change in the calibration constant with increasing temperature appears to be stochastic and unsuitable for correction and is therefore accounted for as an uncertainty. The uncertainties in the calibration constant are treated as uncorrelated and combined through the RSS method. The SE in $C_{cal}$ is found to be 1.7% throughout the calibration temperature range and is assigned as a standard uncertainty. The combined standard uncertainty of FPA calibration is summarized in Table 10.

**Table 10:** Combined uncertainty of the FPA calibration.

Uncertainty Source	Uncertainty (*k =* 1)	Type	Uncertainty in$\;C_{cal}$ (*k =* 1)
TISS radiance	0.75%	B	0.75%
Signal noise, nonuniformity, and linearity correction	1.6%	B	1.6%
Spectral mismatch	1.7%	A	1.7%
Responsivity ($r_{\lambda }^{FPA}\left(\lambda \right)$)	2.0% spectrally	B	0.01%
Lens transmittance ($\tau_{\lambda }^{TEMPS}\left(\lambda \right)$)	0.5% spectrally	B	0.01%
Filter transmittance ($\tau_{\lambda }^{filt}\left(\lambda \right)$)	0.5% spectrally	B	0.01%
Wavelength (λ)	0.5 nm	B	0.3%
	Combined standard uncertainty		2.5%

#### 4.2.6 Retroreflected Emitted Light

The object of measure (the laser-metal interaction scene) emits a potentially significant amount of radiation within the thermometry waveband, 850 ± 20 nm. The radiation emitted from the melt pool is then integrated in the reflectometer. The integrated light then illuminates the scene, which increases the apparent levels of emitted radiance. In this way, the reflectometer surrounding the laser-metal interaction scene causes a bias toward higher measured temperature values.

The hot area is typically approximately 300 µm by 600 µm and can conservatively be estimated to be as hot as 3 000 K. After integrating across the hemisphere and integrating across the waveband of measure, and assuming a conservatively high emissivity value of 0.5, the total emitted power from the melt pool could be as much as 0.17 W.

The total luminous power emitted by the LEDs is on the order of 100 W, which creates an imager signal of approximately 2 000 DL, which is on the same order as the thermography signal near the melting temperature of high-purity Ni used in this study. Therefore, the self-emitted luminous power as a proportion of the LED luminous power provides an estimate of the signal uncertainty generated by retroreflected emitted light. The relative standard uncertainty in the signal due to retroreflected emitted light is estimated to be 0.2%.

#### 4.2.7 Combined Uncertainty of Temperature at the Solidification Point of High-Purity Nickel

The uncertainty in the measured sample temperature at the solidification point of high-purity nickel due to each component is found by perturbing Eq. (34). As described previously, the uncertainties due to spectral effects of the band-pass filter ($\tau_{\lambda }^{filt}\left(\lambda \right)$), TEMPS optics ($\tau_{\lambda }^{TEMPS}\left(\lambda \right)$), and the FPA responsivity ($r_{\lambda }^{FPA}\left(\lambda \right)$) are evaluated by varying the quantities from the minimum to the maximum across the spectral range of interest. The uncertainty of the calibration constant ($C_{cal}$), sample emissivity ($\varepsilon_{samp}\left(\lambda \right)$), and measurement wavelength (λ) are evaluated in absolute terms. The same pixel is evaluated as in Sec. 3.3.5, and the emissivity and its uncertainty from that section are also used. The signal ($S\left(T\right)$) uncertainty incorporates image noise uncertainty, nonuniformity uncertainty, linearization uncertainty, the SE of the signal due to process variability, and the uncertainty due to retroreflected emitted light.

After each change in the measured temperature due to each uncertainty component is evaluated, the results of the RSS method of those values are then taken to determine the combined standard uncertainty. The measurement uncertainties are treated as uncorrelated. It may be noted that the combined effects of the uncertainties are slightly nonlinear, but the asymmetry is small enough to be considered negligible. The probability distribution characterized by the measurement result and its combined standard uncertainty is estimated to be Gaussian. The results are summarized in Table 11.

**Table 11:** Combined uncertainty of temperature at the solidification point of high-purity nickel.

Uncertainty Source	Uncertainty (*k =* 1)	Type	Change in $T_{samp}$ at Maximum Variable Value (°C)	Change in $T_{samp}$ at Minimum Variable Value (°C)
$S\left(T\right)$	1.8%	A	3.2	−3.3
$C_{cal}$	2.5%	B	4.4	−4.5
$\tau_{\lambda }^{TEMPS}\left(\lambda \right)$	0.5% spectrally	B	0.0	0.0
$\tau_{\lambda }^{filt}\left(\lambda \right)$	0.5% spectrally	B	0.0	0.0
$r_{\lambda }^{FPA}\left(\lambda \right)$	2.0% spectrally	B	0.0	0.0
$\varepsilon_{samp}\left(\lambda \right)$	7.0%	B	12	−13.1
λ	0.5 nm	A	0.5	−0.5
Combined asymmetric standard uncertainty (°C)			13	−14

Validation of the temperature measurement and uncertainty will be described in Sec. 4.2.8.

#### 4.2.8 Validation and Discussion: Comparison to the Solidification Point of High-Purity Nickel

The solidification temperature of high-purity (99.998%) Ni is known to be 1 455 °C with a consensus of ±1 °C [42]. The measured value is found to be 1 469 °C, with a standard uncertainty range covering 1 455 °C to 1 482 °C. The measured value is, therefore, in agreement with the table data within the standard uncertainty.

It should be noted that there is potential for Ni to be cooled below its melting point without solidification, which is referred to as “undercooling.” This potential departure from the expected solidification temperature of Ni must be considered in order to use the solidification temperature for validation of the temperature measurements in this work. Under the very steady and spatially isothermal conditions generated by magnetic levitation, 99.999% Ni can be undercooled by more than 180 °C with very slow (quasi-isothermal cooling, on the order of 1 °C/min) temperature decay [43]. The latent heat of fusion is eventually released, and “recalescence” occurs when the temperature temporarily rises toward the expected solidification temperature before falling again.

Nickel can also potentially be subcooled by more than 10 °C in a large laboratory crucible with low vibration conditions and quasi-isothermal cooling [44]. However, under the same conditions with slight mechanical jarring, the observed subcooling is expected to be in the range of 5 °C to 10 °C [44]. The high cooling rate (on the order of 10^6^ °C/s) behind a scanned laser-induced melt pool can lead to constitutional undercooling in front of the solid-liquid transition, which may result in undercooling on the order of 10 °C or more in metal alloys [45], but the high-purity Ni used in this study precludes this undercooling mechanism.

Therefore, the highly dynamic, non-isothermal, laser-induced melt pool of high-purity Ni is not expected to generate substantial undercooling. Furthermore, our spatially resolved temperature profiles do not show any clear evidence of significant undercooling but do show a clear isothermal region that is congruent with the expected profile of a solidification plateau. Therefore, the consensus solidification temperature identified in Bedford *et al.* [42] of 1 455 °C ± 1 °C is used for validation of the temperature measurements in this work. This topic may be further investigated in future work.

It should further be noted that the temperature measurement described thus far in this paper assumes a single-pixel measurement of a uniform scene without consideration of spatial effects. Spatial effects that require signal corrections include stray light, subpixel gradients, and blooming, which will be discussed in Sec. 5. Furthermore, excess radiation emitted, scattered, or reflected from the process by-products above the melt pool are expected to increase the signal and may be expected to cause an erroneously high temperature measurement, which is described in Sec. 6.

## 5 Image Corrections and Uncertainties Associated with Spatial Effects

The topic of Sec. 5 is the image corrections applied to compensate for the spatial effects on FPA signals as well as the estimation of the uncertainties of the corrections. In addition to recording of the melt pool images (on the same high-purity Ni sample used in the single-pixel measurements of Sec. 3 and Sec. 4), three preliminary tests are performed to establish the necessary correction functions. Each one of these tests is described in detail in this section (Sec. 5), but they can be summarized as follows:

(1) A linearization test with an HTBB source over a range of temperatures as described in Sec. 3.3.4.3.3. Data from this test are then used to compose the signal correction factor function.
(2) A stray light and blooming measurement with a fiber-coupled laser source as described in Sec. 5.1.1. Data from this test are used to generate a matrix of erroneous signal due to stray light and blooming, which is then used for image correction.
(3) A knife-edge measurement with a uniform source as described in Sec. 5.1.3.1. The knife-edge data are used to generate an edge spread function (ESF), which is then used to generate a point spread function (PSF).

Each of the three tests should be performed—or confirmed to be accurate—for varying camera settings, melt pool materials, and laser-melting parameters.

The functions established from the preliminary tests are then used for image corrections. These correction factors are contained in $\eta \left(i,j\right)$ and $\zeta \left(i,j\right)$ in the measurement equation in Eq. (23). In the current work, the central 30 frames of the test sequence are used. The first and last 25 frames of the sequence are rejected so that melt pool transients due to laser startup and shutoff are not included in the analysis. The correction operations are then applied to each of the central 30 raw image frames, as will be described in detail in Sec. 5.1, but they are summarized as follows:

(1) Linearization is applied to each pixel of each frame as is described in Sec. 3.3.4.3.
(2) Erroneous signal due to stray light and blooming is subtracted.
(3) A mild smoothing operation is applied to each image.
(4) Deconvolution is performed with the use of the established PSF.

Finally, after each image has been corrected, the pixel mean (in Eq. (27)) forms a single frame representative of the typical corrected signal generated by the process.

The uncertainty associated with each image correction is then evaluated, along with the uncertainty due to the combined effects of process variability and imager noise. The sources of uncertainty in the FPA signal are considered in addition to the non-signal-related uncertainty components in the single-pixel measurement equation (Eq. (34)). The uncertainties due to spatial effects are incorporated into the uncertainties of the spatial-effect correction terms ($\eta \left(i,j\right)$ and $\zeta \left(i,j\right)$) in the spatial-effects correction equation (Eq. (23)) for each pixel of the representative signal frame. Five uncertainty components are identified, as follows:

(1) linearization uncertainty,
(2) uncertainty due to the stray light and blooming correction,
(3) uncertainty due to image smoothing,
(4) uncertainty due to the deconvolution operation,
  (a) uncertainty due to the ESF curve fit and imager noise in determination of the PSF,
  (b) uncertainty due to the deconvolution operation, and
(5) uncertainty due to variability in the (corrected) signal caused by variability of the laser-induced melt pool during the sampling of multiple images and due to imager noise.

Uncertainty components 1 through 4 in the preceding list are uncertainties due to imperfect measurement of the image correction functions, which are applied to each image and then averaged. Therefore, uncertainty components 1 through 4 are not reduced by increasing the sample size (number of frames averaged), and hence are treated as independent of sample size. Uncertainty component 5 is determined by the pixel SE (as in Eq. (26)), and therefore, it does decrease in magnitude with increased sample size. Uncertainty components 1 through 5 are treated as uncorrelated and ultimately combined through the RSS method to generate an uncertainty for each pixel of the representative signal frame.

In this work, blur due to melt pool motion and finite integration time is not considered because the melt pool image is static relative to the laser motion, which generates a quasi-static melt pool reference frame. Although the melt pool does undulate somewhat relative to the laser motion, blur due to melt pool length changes and location variability within the reference frame within the integration time is treated as negligible. The detailed determination and evaluation of each image correction are detailed in Sec. 5.1. Then, evaluation of the correction uncertainties is detailed in Sec. 5.2.

### 5.1 Image Corrections for Spatially Resolved Data Products

#### 5.1.1 Stray Light and Blooming

Stray light is light that passes through the optical system in a manner that is not intended. Imager blooming occurs when a pixel potential well is overfilled and the excess charge bleeds over into adjacent pixels, causing erroneously high signal. Both stray light and blooming tend to increase the FPA signal near high-intensity regions, typically exhibiting an exponential decay in the erroneous signal away from high-intensity regions. Although the TEMPS optical system is designed with best practices to reduce stray light [46], and the imager with a complementary metal–oxide–semiconductor (CMOS) FPA is relatively impervious to blooming, these effects must be measured and compensated for accurate measurements.

Stray light and blooming are measured simultaneously because both have similar causes and effects on the measurements. In order to quantify the combined effect of stray light and blooming, an illumination source other than the melt pool is required to eliminate the optical effects of the laser-melting process variability and by-products. The source chosen for the application is a 6 µm diameter fiber-coupled laser with divergence angle exceeding the acceptance angle of the TEMPS optics. The fiber-coupled laser is located under a 100 µm aperture located at the build plane height so that the aperture is slightly overfilled. Illumination is projected onto the imager through the TEMPS optics system to replicate the melt pool hot spot. The aperture size and intensity are selected to closely mimic the area and intensity of the melt pool hot spot.

A 100 µm aperture is used to approximately replicate the size of the melt pool hot spot, and the laser intensity is set by matching the maximum FPA signal at a SS of 1.25 µs with that of the melt pool, as illustrated in Fig. 23. The imager SS is then increased to 98.3 µs to match the SS used in the emissivity and temperature measurements. Similar to the thermal imaging of a melt pool, this creates a significant saturated region around the hot spot. The ratio of the low to high SS values is approximately proportional to the excess radiant flux passing through the TEMPS optics and focused onto the FPA. This means that there is on the order of 100 times more radiant flux passing through the optics and onto the FPA within the saturated region than the intensity levels in the nonsaturated regions of interest. This can produce a significant potential for erroneous signals due to stray light and blooming.

**Fig. 23:** Images from a stray light and blooming experiment compared with melt pool images of a comparable area and intensity at two SS values. The images are the same scale. **Table:** 

	Melt pool Image	Laser Image
SS = 1.25 µs (nonsaturating)	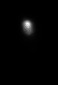	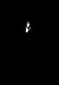
SS = 98.3 µs (saturating)	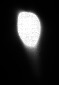	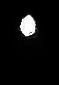

**Fig. 24:**
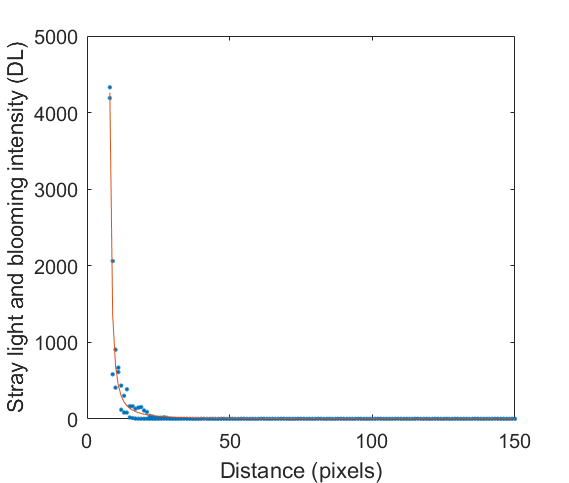
Erroneous signal due to stray light and blooming.

As shown in Fig. 24, two representative cross sections of the linearized FPA signal due to stray light and blooming are curve fit to establish a relation between erroneous signal intensity and distance from the hot-spot center. Only data from pixel 8 (at 48 µm radius, the approximate radius of the aperture) and above are used for the curve fit.

Equation (41) is the resulting fit, with R^2^ = 0.963, of the erroneous signal due to stray light and blooming (*S_SB_*), where *r* is the distance in pixels:

**Table:** 

	$S_{SB}=4260{\left(r-7\right)}^{-1.65}$	(41)

**Fig. 25:**
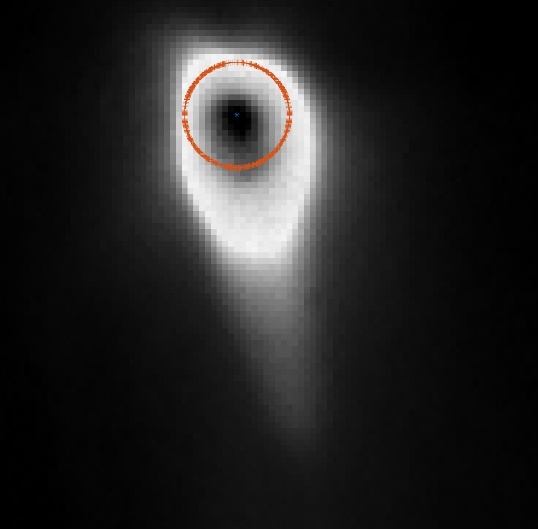
Melt pool signal generated with 1.25 µs SS subtracted from that of the melt pool signal generated with 98.3 µs SS, along with the 100 µm diameter of the aperture (encircled). The hot spot is located by the maximum intensity of the hot spot with 1.25 µs SS.

The center of the hot spot is then located based on measurements from a representative melt pool. Independent measurements are taken with 1.25 µs SS and 98.3 µs SS. The hot spot is located by the maximum intensity with 1.25 µs SS. For illustration, the melt pool signal generated with 1.25 µs SS is subtracted from that of the melt pool signal generated with 98.3 µs SS, and the result is shown in Fig. 25, along with the 100 µm diameter of the aperture.

The distance from the center of each pixel to the hot-spot center is then calculated. The erroneous signal due to stray light and blooming is calculated using Eq. (41) based on the distance from the hot-spot center (*r*). Finally, the erroneous signal is subtracted from each melt pool image of interest. The pixels in the saturated region are left at the original saturated DL. After subtraction of the signal due to stray light and blooming, a mild smoothing operation is applied to images, which will be discussed in Sec. 5.1.2.

#### 5.1.2 Image Smoothing

A mild smoothing filter is applied to each test image after the stray light and blooming subtraction. Smoothing the images reduces the effects of noise on the deconvolution. The smoothing filter is based on nearest-neighbor averaging with one adjacent pixel on either side, top, and bottom. Finally, it is confirmed that the smoothing operation does not introduce a systematic signal bias by subtracting the original image from the smoothed image and averaging across the frame. The resulting average signal difference results in a negligible average bias of less than 1% of a digital level at each pixel. Image deconvolution is performed after smoothing.

#### 5.1.3 Image Deconvolution

Deconvolution is an image correction operation intended to reconstruct an image into its original form prior to blur induced by unavoidably nonideal optics and the finite pixel size of FPAs. The multistep deconvolution approach taken here is based on that of Lane and Whitenton [47] and ISO 12233:2017 [48]. First, a knife-edge measurement is made to establish an ESF. The ESF is then transformed into a PSF, which can be thought of as a “deblurring kernel.” Finally, the images are deconvolved. The details of each operation are discussed in the remainder of this section (Sec. 5.1.3).

##### 5.1.3.1 Knife-Edge Measurement

Use of an ESF measurement is a practical approach to determination of the PSF, because use of a true point source is experimentally difficult, if not impossible [48]. Use of an ESF to determine the PSF reasonably assumes that the response of the optical system is rotationally symmetric.

**Fig. 26:**
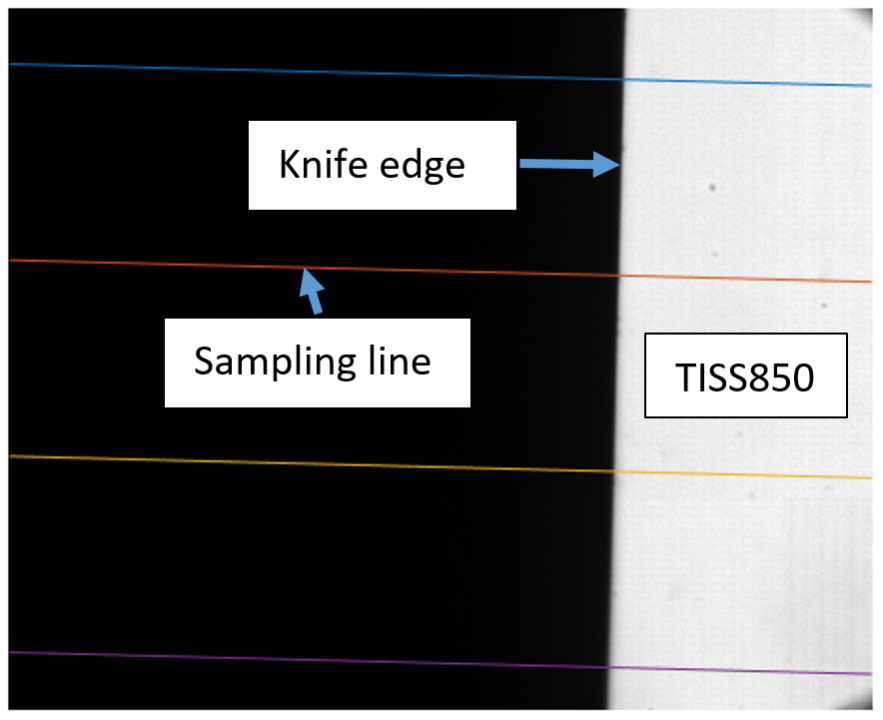
Near-vertical knife-edge measurement image with four sampling lines.

To establish the ESF, a thin, opaque, and straight edge is placed to partially cover the aperture of the TISS850 set at a radiance temperature of 1 600 °C at the build plane. The edge is placed a few degrees from the vertical so that the edge is not perfectly aligned with the pixel array [47, 48]. Sampling lines are then taken perpendicular to a curve fit of the maximum image gradient to mark the edge between high and low DL, as shown in Fig. 26. The image data shown in Fig. 26 were taken with resolution of 6.0 µm per pixel through the TEMPS optics and the imager is set at a SS of 98.3 µs. Determination of the ESF from this measurement is described in Sec. 5.1.3.2.

##### 5.1.3.2 Edge Spread Function Determination

The four sampling lines shown in Fig. 26 are found to be negligibly different, and so one is treated as representative of the image profile. The representative profile is then normalized to have a peak intensity of unity and is supersampled with four samples per pixel along the length of the array, per ISO 12233:2017 [48]. The data are then centered on zero based on the maximum gradient of the profile. In sampling with one-, two-, and three-component error function fits, it is found that the most appropriate fit is with the two-component error function, shown in Eq. (42).

**Table:** 

**Fig. 27:**
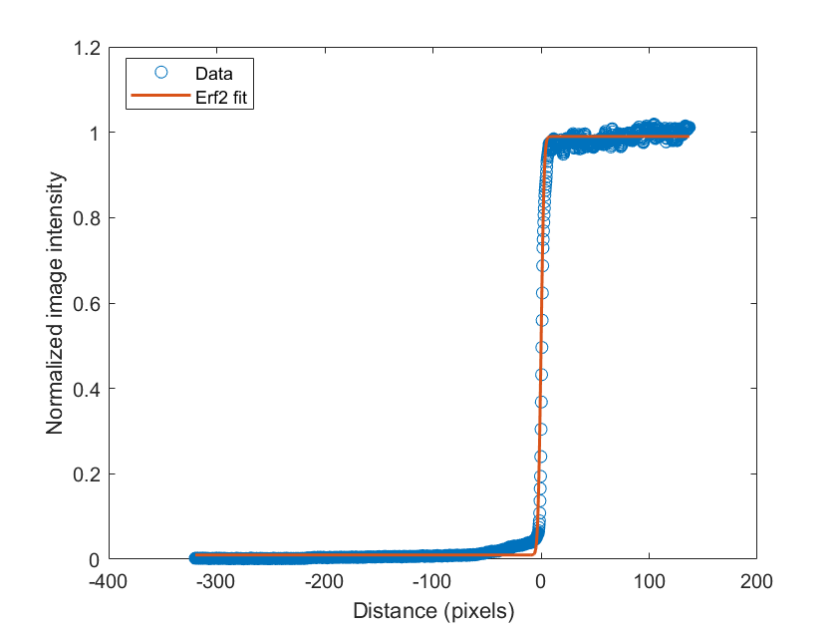
Supersampled ESF array with a two-component error function curve fit.

	$ESF=a_{1}\mathrm{erf}\frac{x}{b_{1}}+a_{2}\mathrm{erf}\frac{x}{b_{2}}+0.5$	(42)

where *x* is distance in pixels, $a_{1}=-68.55$, $b_{1}=\;3.184$ [pixels], $a_{2}=69.04$, and $b_{2}=\;3.184$ [pixels]. The supersampled knife-edge measurement data along with the Eq. (42) curve fit are shown in Fig. 27. The curve fit has *R*^2^ = 0.998. Determination of the PSF from the ESF will be described in Sec. 5.1.3.3.

##### 5.1.3.3 Point Spread Function Determination

The PSF is obtained from the ESF using an Abel transform as described in Ref. [47]. The corresponding PSF is shown in Eq. (43).

**Table:** 

**Fig. 28:**
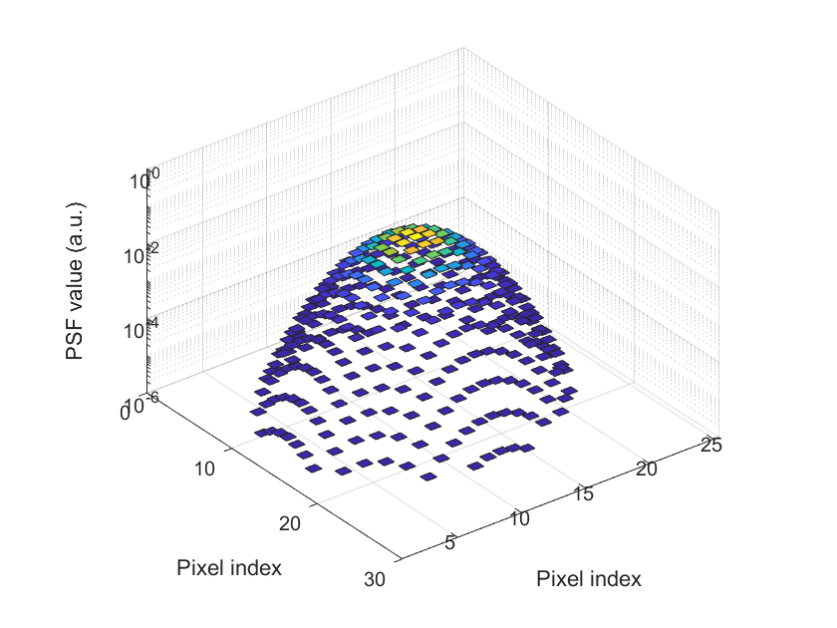
PSF array based on the knife-edge measurement.

	$PSF=\frac{2}{\pi }\frac{a_{1}}{b_{1}^{2}}\exp\left(-\frac{r^{2}}{b_{1}^{2}}\right)+\frac{2}{\pi }\frac{a_{2}}{b_{2}^{2}}\exp\left(-\frac{r^{2}}{b_{2}^{2}}\right)$	(43)

where *r* is the radial distance in pixels, and $a_{1}$, $b_{1}$, $a_{2}$, and $b_{2}$ are the values determined in Eq. (42). The value of the PSF array is calculated at each supersampled pixel location using Eq. (42) and then averaged across the 4 × 4 supersampled pixel to determine the PSF value at each pixel. The PSF resulting array is 25 pixels × 25 pixels in order to cover four orders of magnitude of intensity from the PSF center to the perimeter. Finally, the volume under the PSF array is normalized to unity. The PSF array used for deconvolution is shown in Fig. 28. Deconvolution is discussed in Sec. 5.1.3.4.

##### 5.1.3.4 Deconvolution

Each of the central 30 images of the test is individually deconvolved to allow for later uncertainty analysis of the deconvolved image transient variability. Each image first has stray light and blooming removed, and then it is smoothed, as discussed in the preceding sections, before deconvolution. The PSF array is then used to deconvolve each image with the MATLAB deconvlucy function, which is based on the iterative Richardson-Lucy method [49, 50].

Figure 29a shows the average image after subtraction of the stray light and blooming signal and smoothing. It can be observed from Fig. 29b that the same image is “sharpened” after deconvolution. The sharpening causes an apparent narrowing of the melt pool and steepening of the signal gradients on the left and right sides, as well as at the nose. Toward the end of the tail, the local signal gradient becomes shallower and longer along the length of the melt pool, but steeper in the transverse direction across the tail. This effect of deconvolution becomes more intuitively understandable if Fig. 29a is thought of as a blurred version of Fig. 29b (instead of the other way around in reality)—the high local gradients transverse to the tail cause a significant blurring effect on the shallower gradients along the length of the tail.

Deconvolution also accentuates two signal features to the lower left and upper right of the melt pool nose. These anomalously high signals at a relatively large distance transverse to the laser scanning direction are believed to be due to radiation emitted by the plume of laser-melting by-products. The effects of this radiation on emissivity and temperature measurements will be discussed in Sec. 6. The following section (Sec. 5.1.4) is a summary of the effects of the signal corrections discussed in this section (Sec. 5.1).

**Fig. 29:** (a) Image before deconvolution and (b) image after deconvolution. **Table:** 

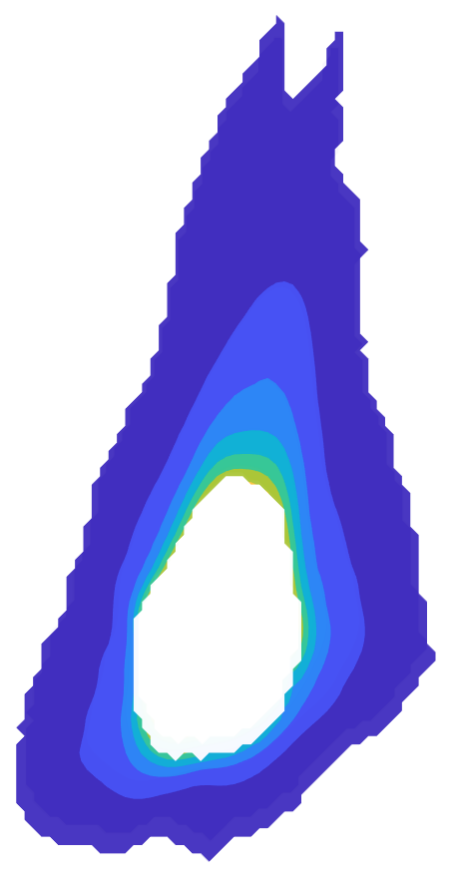	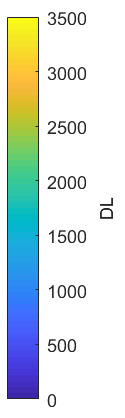 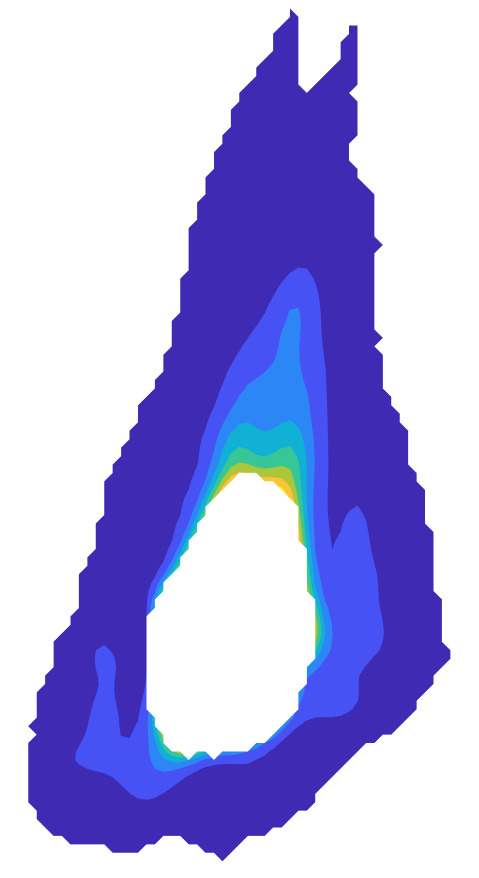

#### 5.1.4 Summary of the Signal Corrections

The effect of each image correction operation is shown by a central cross-sectional profile along the length of the melt pool, with and without LED illumination, in Fig. 30. Each profile is taken from a pixel mean frame of the central 30 frames of the test at each correction step. Starting with the linearized signal, the stray light and blooming subtraction operation reduces the signal at the nose of the melt pool because of its proximity to the hot spot, but it has a very small effect on the signal at the tail because of its larger distance from the hot spot. The smoothing operation has very little effect on the signal levels with LEDs off, but it significantly reduces the apparent pixel-to-pixel noise caused by LED illumination.

Deconvolution tends to have the most notable effect on the signal levels, both at the nose and the tail of the melt pool. Deconvolution sharpens the profile, reducing the signal at areas of high curvature, and steepening the profile at areas of high (three-dimensional) gradients. This can be observed at the nose and the tail of the melt pool at pixel 15 and pixel 57, respectively, in Fig. 30. The most significant variation in the central cross-sectional profile due to deconvolution occurs at the tail, which generates a “solidification plateau.” At this solidification plateau from pixel 63 to pixel 68, the signal is flat, indicating an apparently isothermal solidification region as the laser heat source moves. This isothermal region is expected in the solidification of high-purity metals (like the 99.998% Ni used here), because the fixed-point temperature at which a phase change occurs is maintained as the material dissipates the latent heat of fusion at the solidification temperature. The solidification region is made significantly more apparent in the signal by deconvolution. Deconvolution also causes an apparent dip in the signal at pixel 12 with the LEDs on, which is likely due to erroneous signal values in the saturated region from pixel 19 to pixel 46. The signal uncertainty associated with each signal correction operation will be discussed in Sec. 5.2.

**Fig. 30:** The central cross-sectional profile along the length of the melt pool showing the effect of each image correction without LED illumination (top) and with LED illumination (bottom). Each profile is taken from a pixel mean frame of the central 30 frames of the test at each correction step. The melt pool nose is on the left, and the tail is on the right, with the scan direction from right to left. SL is stray light, and B is blooming. **Table:** 

LEDs off 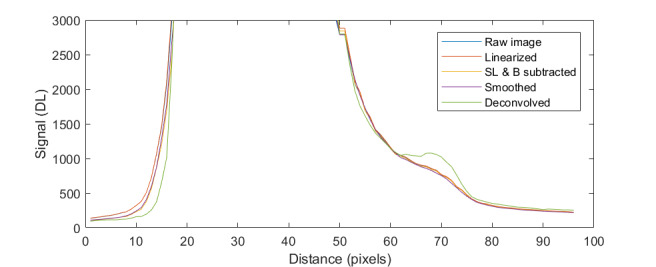
LEDs on 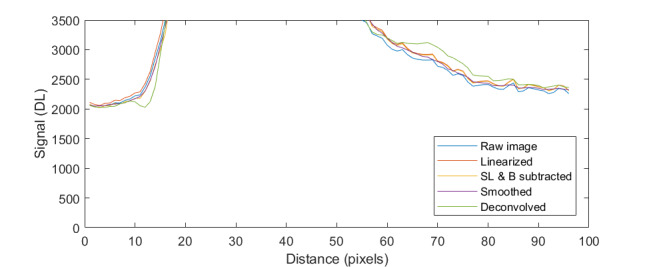

### 5.2 Uncertainty Evaluation of Image Corrections

As shown in this Sec. 5 so far, the signal is the only parameter in need of correction due to spatial effects and is therefore the only parameter in the measurement equation that incurs additional uncertainty due to spatial effects. The signal uncertainty incurred by each operation on each pixel is calculated for the corrected images. The details of evaluating uncertainty are described in the remainder of Sec. 5.2.

#### 5.2.1 Linearization Uncertainty

As described in Sec. 3.3.4.3, the uncertainty of the signal linearization operation resulting from the linearization measurement and data analysis procedure results in a typical uncertainty of 1.6%. It should be noted that this uncertainty is evaluated based on signal values after deconvolution, which inherently assumes that the linearization uncertainty transforms linearly through the deconvolution operation. Because the linearization uncertainty is a relatively small component of the total uncertainty, this is believed to be a reasonable estimation.

#### 5.2.2 Stray Light and Blooming

The range-normalized root mean square error (RMSE) of the curve fit applied to the erroneous signal due to stray light and blooming shown in Fig. 24 is 1.7%. This is used as the uncertainty of the curve fit of the stray light and blooming signal matrix combined with the uncertainty due to noise in the knife-edge measurements. As described in Sec. 3.3.4.3, the uncertainty of the signal linearization operation is 1.6%, which is an additional uncertainty in the laser illumination test data. The two uncertainties are combined by the RSS method, and the combined uncertainty of the stray light and blooming correction is then 2.3% of the DL of each pixel of the erroneous signal due to the stray light and blooming matrix. The erroneous signal is subtracted from each of the 30 central frames of the test, and the resulting signal uncertainty due to the correction uncertainty is evaluated after pixel averaging of the frames to determine the representative melt pool signal.

#### 5.2.3 Image Smoothing

As stated previously, it is confirmed that the smoothing operation does not introduce a systematic bias. This is shown by subtracting the original image from the smoothed image and averaging across the frame, resulting in a negligible bias of less than 1% of a digital level per pixel. Therefore, uncertainty due to the image smoothing is assumed to be negligible.

#### 5.2.4 Signal Uncertainty due to PSF Uncertainty

The uncertainty in the PSF is due to the uncertainty in establishing the ESF by curve fitting of the empirical data. The linearization operation of the knife-edge data leads to an initial signal uncertainty component of 1.6%. The curve fit leads to an uncertainty component of 2.0%, which is the RMSE normalized by the range. In order to establish a PSF at the uncertainty extremes, the ESF is scaled in the positive and negative direction by the combined uncertainty of 2.6%, and the PSF is reevaluated. From this, new constants $a_{1}$, $b_{1}$, $a_{2}$, and $b_{2}$ for Eq. (43) are found.

**Fig. 31:**
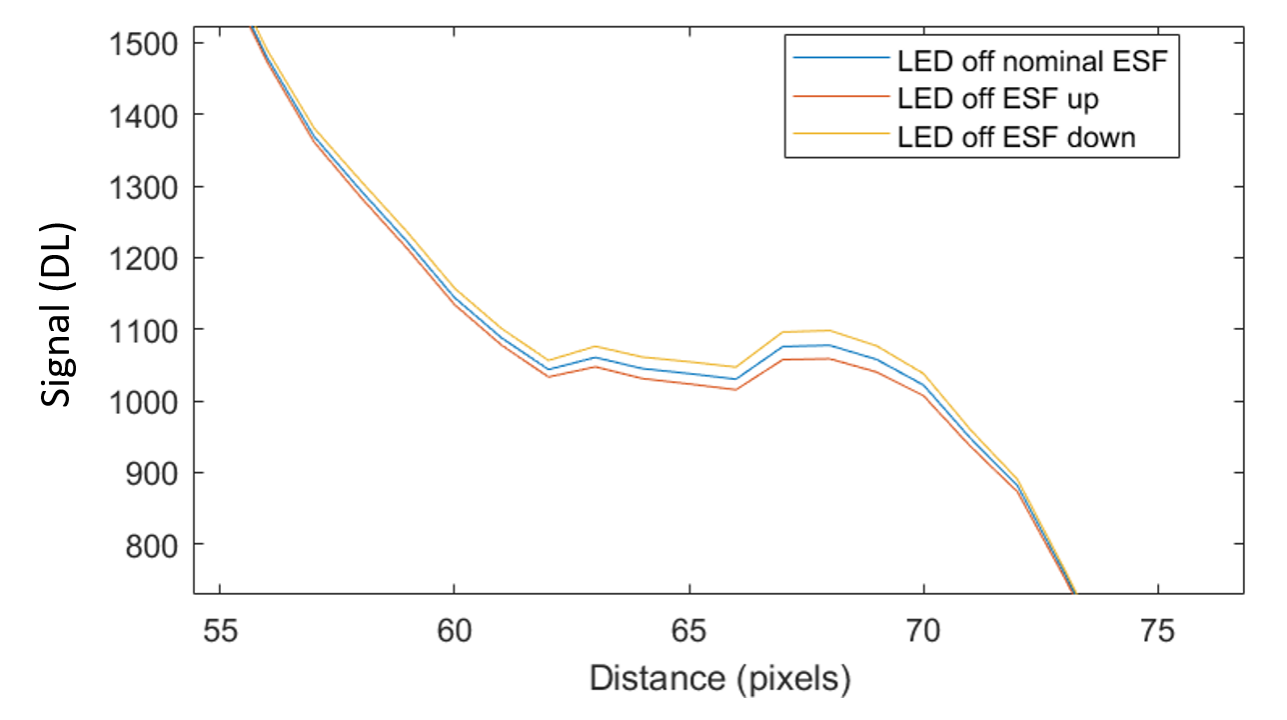
Deconvolved signal profiles at the solidification plateau evaluated with the nominal PSF and at the standard uncertainty extremes. ESF up indicates that the ESF curve fit is 102.6% of its nominal value, and ESF down indicates the ESF curve fit is scaled by 97.4% of its nominal value when determining the PSF.

The method described in Sec. 5.1.3.3 is then used to establish two PSF arrays at both extremes of the uncertainty range due to the ESF. The images are then deconvolved using the method described in Sec. 5.1.3.4 with each PSF. The change in signal due to either extreme of the PSF values is nearly symmetric, as illustrated in Fig. 31 at the solidification plateau region where the largest change is observed. The maximum signal change is less than 2% at pixel 67 and pixel 68. The pixel mean of the deconvolved image produced by the addition of the uncertainty to the PSF is then subtracted from the nominal pixel mean of the deconvolved image to establish an uncertainty matrix due to error in the PSF determination, which is used as the standard uncertainty of the signal due to the establishment of the PSF.

#### 5.2.5 Deconvolution

**Fig. 32:**
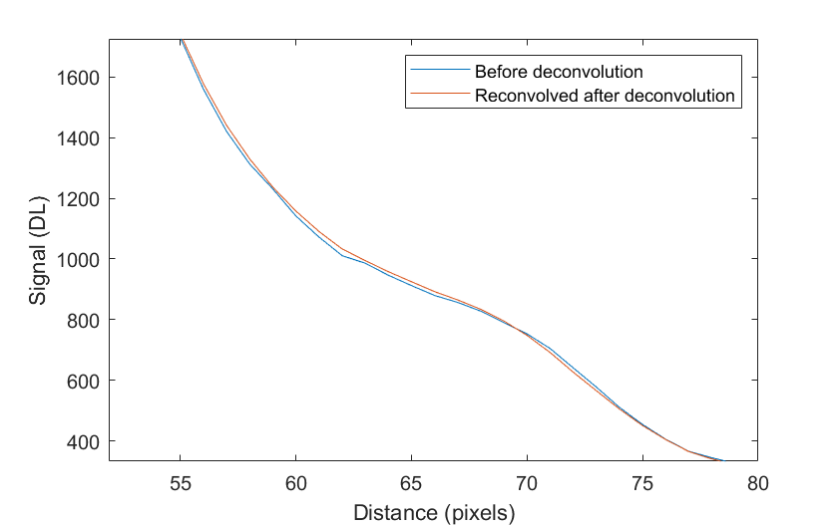
Comparison between the pixel mean image profile before deconvolution and after deconvolution and reconvolution at the solidification plateau.

The Richardson-Lucy algorithm is among the most robust deconvolution algorithms, but it is an iterative algorithm designed to converge on the most likely reconstructed signal values [51, 52]. Conversely, convolution is a destructive forward calculation that can be done with very little error. A comparison between the pixel mean image profile before deconvolution and after deconvolution and reconvolution at the solidification plateau is shown in Fig. 32. It is observed that the discrepancy is generally quite small, on the order of 1% to 2% at most pixel locations. The reconvolved image is subtracted from the unconvolved image at each of the central 30 frames, and then the pixel mean is evaluated to determine the average discrepancy at each pixel. This pixel mean discrepancy is used as the standard uncertainty due to the deconvolution operation.

Deconvolution error also occurs due to the saturated region of the image, in which the signal values are no longer proportional to the local radiant flux. In order to reduce the error associated with deconvolution of false signal values, a five-pixel border around the saturated region is discarded. Within the radius of five pixels, approximately 97% of the PSF volume is contained, and therefore false signal values should have a negligible effect outside of that radius. In future work, signal values may be extrapolated to five pixels within the saturated region to reduce the number of discarded pixels. Alternatively, a further improvement may be implemented by measuring the melt pool at varying SSs to increase the useful dynamic range of the measurement, eliminating any saturated signal values. Further, a well-controlled and characterized replicate of the melt pool radiance profile (generated perhaps by a mask with spatially varying transmittance and illumination by a steady source) may be used to experimentally characterize the uncertainty associated with the deconvolution signal correction.

#### 5.2.6 Process Variability and Signal Noise

As stated previously, the corrections are applied to each of the central 30 frames. The pixel mean is then evaluated to determine a characteristic profile. The pixel SE is also evaluated (as in Eq. (26)) to determine the standard uncertainty resulting from the combined effects of process variability and imager noise. The magnitude of this standard uncertainty component can be reduced by increasing the sample size or the number of frames of the measurement sequence.

#### 5.2.7 Combined Standard Uncertainty of Signal Values

As described in the preceding sections, each of the five uncertainty components is calculated independently at each pixel to determine the local uncertainty of the signal of each pixel. Signal uncertainties due to linearization, the stray light and blooming correction, the PSF determination, and the deconvolution process are due to the imperfect measurement and evaluation of the image correction functions. Therefore, these uncertainties are not reduced by increased sample size (number of frames evaluated). Signal uncertainty due to process variability and imager noise is obtained from pixel signal SE, which will decrease in magnitude with increased sample size. The five uncertainty components are treated as uncorrelated and combined through the RSS method to generate a combined signal uncertainty for each pixel of the pixel mean melt pool corrected image.

Image data from the reference mirror (with the LEDs on and off) do not require a stray light and blooming correction or deconvolution. Therefore, the combined uncertainty of the reference mirror images has only three components: linearization uncertainty, FPA nonuniformity, and pixel noise, which are evaluated as described in the preceding sections.

**Fig. 33:**
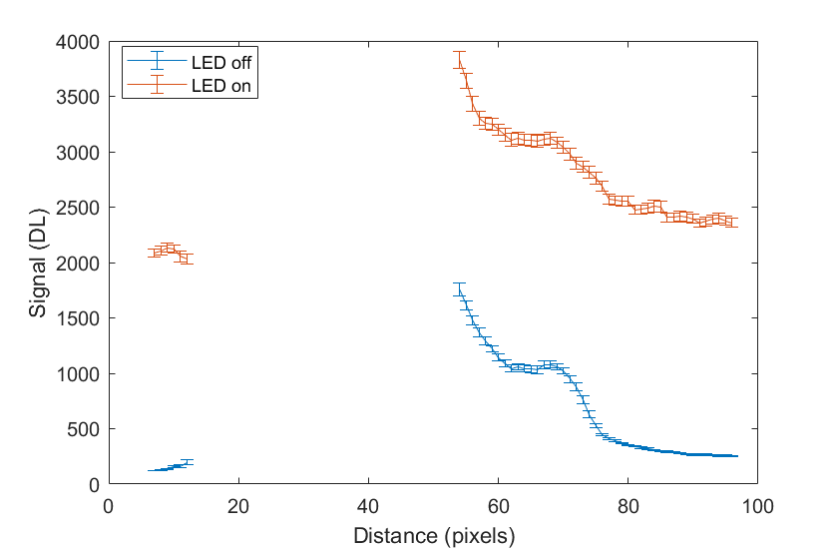
Corrected signal cross sections and combined standard uncertainties. The data parameters included laser power of 250 W, scan speed of 1 000 mm/s, D4σ spot size of 65 µm, image rate of 10 000 Hz, and 98.3 µs SS. Melt pool nose is on the left, and tail is on the right, with the scan direction from right to left.

Central cross-sectional profiles of the corrected signal and standard uncertainty with and without LED illumination are shown in Fig. 33. As described previously, the signal uncertainty varies with each pixel, but typical uncertainty values are in range of approximately 1% to 3%. It can be observed that the signal value at the nose of the melt pool decreases from pixel 10 to pixel 12 with the LEDs on, but it increases in the same pixel range with the LEDs off. The dip in signal appears to be an artifact of deconvolution of the LED on images, which may be due to the close proximity to saturated signal values. Approaches to rectify this apparent error in the future are described in Sec. 5.2.5.

The solidification plateau is evident from pixel 63 to pixel 68 with the LEDs on and off, which illustrates the apparent important effect of image correction, especially deconvolution, in observation of the melt pool temperatures. In the solidified region of pixels 75 and more, the signal is quite smooth with LEDs off, whereas with the LEDs on, the signal appears unsteady. These unsteady signal values with LED illumination may be due to surface features (chevron patterns) created by the laser-melting process, which change the local reflectance/emissivity. The results of emissivity and true temperature measurements from these corrected signal values are discussed in Sec. 6.

## 6 Measurement Uncertainty of Spatially Resolved Temperature and Emissivity

The pixel mean values and standard uncertainties of the corrected signal established in Sec. 5 form representative profiles of the HAZ with and without LED illumination. These corrected signal values and uncertainties are then evaluated at each pixel to calculate the emissivity and true temperature. The measurement equations and resulting standard uncertainty in emissivity (Eq. (24)) and the true temperature (Eq. (34)) are evaluated in the same fashion as is described in Sec. 3 and Sec. 4, respectively, but with use of the spatial-effects-corrected signal and its uncertainty at each pixel. The measurand (signal) distribution is assumed to be Gaussian. As discussed in Sec. 3 and Sec. 4, the measurement uncertainties are negligibly asymmetric, and so they are treated as symmetric here. In the evaluation of the uncertainty of the measured emissivity and temperature due to each uncertainty component, the direction (positive or negative change of each variable by the magnitude of the standard uncertainty) that causes the maximum cumulative change of the measured value is used for the evaluation of the associated uncertainty of that component.

### 6.1 Emissivity and Uncertainty

The resulting emissivity values are shown in Fig. 34a, and the associated relative standard uncertainties are shown in Fig. 34b. Figure 34c shows a central cross-sectional profile of the emissivity and uncertainty along the melt pool corresponding to the dotted line shown in Fig. 34a and Fig. 34b with nose on the left and tail on the right.

Beginning with Fig. 34a, it can be observed that emissivity values of more than 0.42 (emissivity is unitless as described in Sec. 1.1) occur near the hot spot. This is likely caused by the vapor depression generated by a hot vapor jet emanating from the laser-metal interaction area. The resulting depression in the molten metal becomes a trap for illumination light by multiple reflections, and therefore decreases the local reflectivity, increasing the local emissivity.

**Table:** 

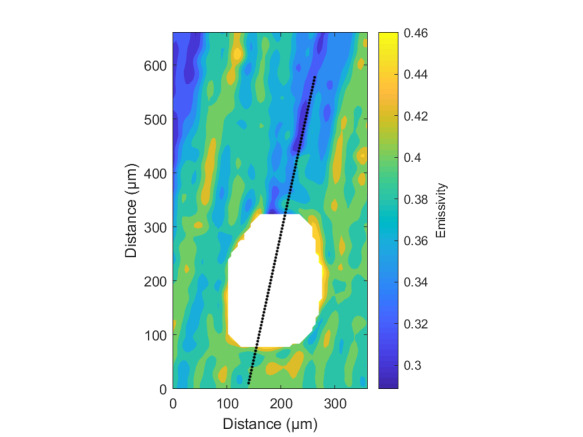	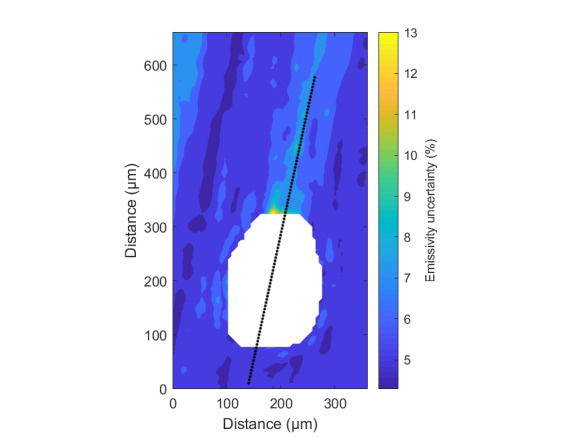
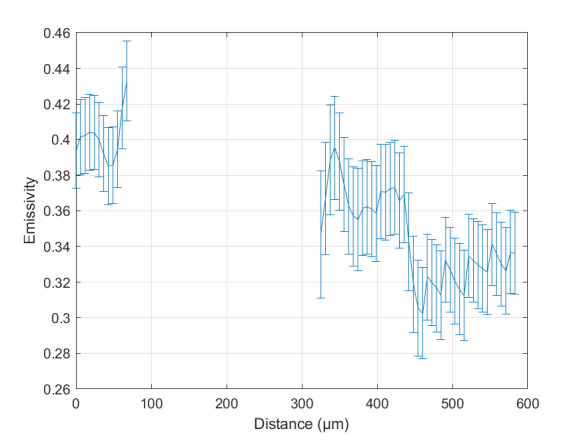	

**Fig. 34.** (a) The emissivity map of a melt pool, (b) the relative combined standard uncertainty of the emissivity map, and (c) the central cross-sectional profile of the emissivity and the uncertainty along the dotted line shown in (a) and (b) (the nose on the left and the tail on the right). The data parameters include laser power of 250 W, scan speed of 1 000 mm/s, D4σ spot size of 65 µm, image rate of 10 000 Hz, and 98.3 µs SS with a 99.998% Ni plate. The standard uncertainty of the dimensional coordinates is estimated to be ±6 µm.	

As stated previously, the signal with LED illumination changed significantly at the nose of the melt pool due to the deconvolution operation. Future work will determine if this change, and the resulting rise in measured emissivity at the nose of the melt pool, is accurate or a shortcoming of the deconvolution of the saturated signal values.

The emissivity of the virgin surface of the nickel plate, which is ground randomly with 320 grit sandpaper, and which can be observed to the left, front, and right of the melt pool, has a measured emissivity of approximately 0.39 with standard uncertainty of approximately 0.02. The lowest emissivity values are observed in the molten and solidified material behind the tail, with typical values in the range of 0.31 to 0.35. These areas that have transitioned to liquid (and/or back to solid) have a higher reflectance because the surface grinding marks have been eliminated, resulting in a lower emissivity. A region of emissivity ranging from approximately 0.30 to 0.32 can be observed in the upper-left corner, which is a cooled (solidified) track that was melted in a previous test.

The highest relative combined standard uncertainty of the emissivity occurs in the tail of the melt pool, as well as in the solidified track in the upper-left corner. Typical relative standard uncertainty values range from 7% to 8%. Lower relative standard uncertainties are observed in the virgin surface, with values typically ranging from 5% to 6%. The measurement uncertainty of the reflectance is generally proportional to the reflectance value, and, therefore, it is expected that the emissivity uncertainty will be greater in the lower emissivity regions.

The location of the solidification plateau is evident in Fig. 34 from pixel 63 to pixel 68, which corresponds to a distance of 380 µm to 411 µm in Fig. 34c. The measured value of the near-normal spectral emissivity near the solidification temperature of 99.998% Ni (1 455 °C) ranges from approximately 0.36 to 0.37 in that region with a standard relative uncertainty of approximately 8%. Published data on a similar material at 1 491 °C resulted in a normal spectral emissivity of approximately 0.36 at 850 nm [34]. Therefore, under the conditions of comparison (Ni melted with a rapidly scanned laser), the emissivity measurement approach developed here is in good agreement with published values.

### 6.2 Temperature and Uncertainty

The resulting temperature values are shown in Fig. 35a, and the associated standard uncertainty is shown in Fig. 35b. Figure 35c shows a central cross-sectional profile of the temperature and uncertainty along the melt pool corresponding to the dotted line shown in Fig. 35a and Fig. 35b, with the nose on the left and the tail on the right. As described previously, emissivity uncertainty is the largest single component contributing to the temperature uncertainty, and it can be observed that the area of highest emissivity uncertainty in Fig. 34b in the melt pool molten tail corresponds to the location of highest temperature uncertainty in Fig. 35b.

In Fig. 35c, it can be observed that temperatures at the solidification plateau from a distance of 380 µm to 411 µm are approximately constant, with values ranging from 1 499 °C to 1 502 °C. The average of the six adjacent pixels with approximately constant temperature is 1 500 °C with standard uncertainty values of approximately 16 °C. The solidification temperature of high-purity Ni is known to be 1 455 °C ± 1 °C [42]. The expanded (*k* = 2) uncertainty of the measured temperature covers a range of 1 468 °C to 1 532 °C at the solidification plateau. Hence, the expanded uncertainty range does not contain the expected solidification temperature of high-purity Ni.

The apparently high measured temperature is likely primarily due to process by-products. The by-products generally originate from evaporation of the metal. Evaporation occurs when the applied laser power, scan speed, and spot size deliver a sufficiently high energy for the phase change from liquid to vapor to occur. Evaporation of the metal causes a vapor jet away from the surface of the laser incidence. The ejected vapor then cools rapidly and can condense in a cloud above the melt pool. This can result in ultrafine condensate particles of 80 nm to 100 nm diameter with a density of approximately 10^10^ particles per cubic centimeter [53]. The nanoscale particles may self-emit within the waveband of measure while also potentially scattering and reflecting radiant flux from the melt pool self-emission. These effects appear to result in excess radiance from the area on and around the HAZ, which would result in erroneously elevated measured temperatures.

**Table:** 

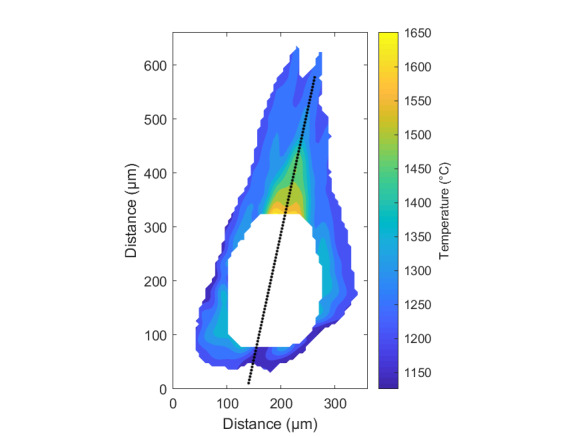	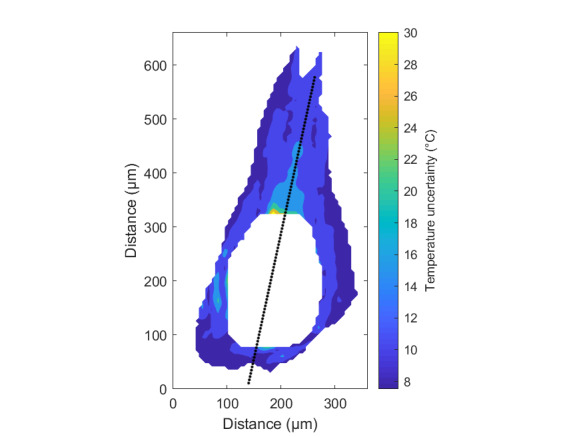
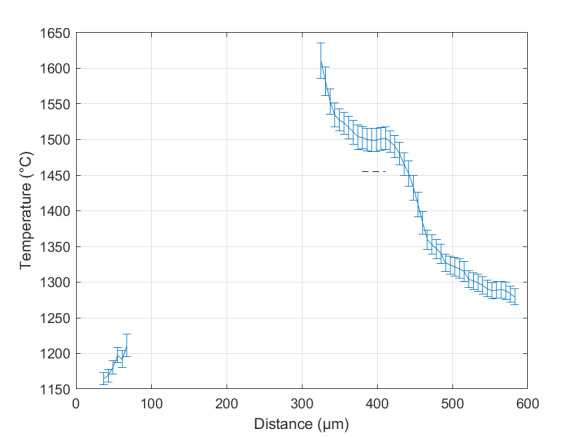	

**Fig. 35.** (a) The temperature map of a melt pool, (b) the combined standard uncertainty of the temperature map, and (c) the central cross-sectional profile of the temperature and the uncertainty along the dotted line shown in (a) and (b) (the nose on the left and the tail on the right). The data parameters include laser power of 250 W, scan speed of 1 000 mm/s, D4σ spot size of 65 µm, image rate of 10 000 Hz, and 98.3 µs SS with a 99.998% Ni plate. The standard uncertainty of the dimensional coordinates is estimated to be ±6 µm.	

The effects of process by-products are most apparent at the left and right sides of the melt pool nose in Fig. 35a. At the left side (at a horizontal position of 95 µm and a vertical position of 114 µm) and at the right side (a horizontal position of 282 µm and vertical position of 210 µm) of the melt pool nose, the measured temperatures exceed 1 350 °C. It is unlikely that the surface temperatures are elevated to this temperature in these areas, so it is far more likely that the measured radiance in these locations is from a reflection off of the virgin surface caused by process by-products above the melt pool. The apparently erroneous signal measured adjacent to the melt pool on the left and right sides is in excess of 500 DL. An excess signal generated by process by-products equivalent to 200 DL would generate a temperature measurement bias of approximately 40 °C high at the solidification plateau. It should be noted that the excess radiation exists with and without LED illumination, which causes this effect to largely cancel out in the emissivity measurement equation, and results in a measured emissivity comparable to that of published values in this paper. Attenuation and/or scattering of LED illumination light may cause a discrepancy in measured emissivity values under different process conditions, however. Therefore, the measurement of, and correction for, the erroneous signal that is generated by process by-products is the subject of future research for improved emissivity and temperature measurement accuracy.

## 7 Conclusions and Further Research

### 7.1 Summary and Conclusions

We describe the concept, the first principles measurement approach, and the data analysis involved in the measurement of the spatial distribution of the temperature of the metal surface of a laser-induced melt pool for application to LPBF processes. The measurement capability developed here sets the foundation for establishing reference thermographic data for LPBF process modelers, as well as multispectral thermal signature data of standard LPBF tracks to support process monitoring and cross-platform process equivalence. This paper also seeks to establish appropriate nomenclature for accurately describing the object of measure, measurement approach, and results.

In this work, a unique high-temperature reflectometer was successfully implemented and characterized. The developed reflectometer is, to the best of our knowledge, the first to use an integrating hemispherical illumination setup, which proved to be a practical approach. The high-intensity hemispherical illumination (850 nm band) used in this study produces an apparent radiance temperature of the target (with emissivity of 0.5) equal to 1 610 °C. This enables reflectometry of high-temperature targets that produce significant self-emission. No performance degradation of the reflectometer is observed due to damage by the high-intensity reflected laser light from the laser-melting process or from contamination by the laser-melting process by-products. An extensive set of emissivity measurement uncertainty components is identified, though not all of the identified uncertainties are fully quantified. For these latter type B uncertainty components, approaches are identified for better quantifying them if required in the future. All of the emissivity measurement uncertainty components that are strictly estimates are given conservatively high values so as to avoid underestimating the magnitude of the combined emissivity measurement uncertainty.

The temperature calibration established here is traceable to the ITS-90. Temperature measurement uses a single spectral band centered at 850 nm with a half-height width of 40 nm, and it results in measurement of temperatures in the range from approximately 1 150 °C to 1 650 °C. The NIST Additive Manufacturing Metrology Testbed (AMMT) high-temperature blackbody (HTBB) is estimated to have a standard relative radiance uncertainty of 0.6%, which translates to a standard radiance temperature uncertainty of 0.6 °C at 1 000 °C and 1.2 °C at 1 500 °C.

A nonthermal, band-limited calibration source (TISS850) was developed for calibration of the FPA for radiance-based high-temperature metrology within the AMMT build chamber (transferred from primary sources to the HTBB and then to the TISS850), because compact and accurate thermal sources are not readily available for the requirements of this application. The general design of the source is described, as well as the thermal stabilization approach for the LED sources. After accounting for all known uncertainties throughout the calibration chain, the calibration constant of the FPA that is coaxially aligned with the heating laser is estimated to have a standard relative uncertainty of 2.5%.

The high intensity and steep gradients of the melt pool scene require image correction operations to correct for erroneous signal generated by spatial effects. Stray light and blooming correction are described, as well as the uncertainty associated with the correction. The uncertainty in the deconvoluted signal due to the uncertainty in measurement of the edge spread function that is used to establish the point spread function is evaluated. The uncertainty in the deconvoluted signal due to the error associated with the iterative deconvolution algorithm is also estimated. The image deconvolution is found to significantly accentuate the solidification plateau of the melt pool, which has important ramifications for the measurement of surface temperatures and cooling rates.

The measured emissivity and solidification temperature are then compared with established values for 99.998% Ni. The bare plate was randomly ground with 320 grit sandpaper, and the build plate is at room temperature. The test parameters include laser power of 250 W, scan speed of 1 000 mm/s, D4σ spot size of 65 µm, and the built plate at room temperature. The imager frame rate is 10 000 Hz, the imager integration time is 98.3 µs, and the pixel size is 6.0 µm per pixel. The laser incidence angle is approximately 8° from normal. The local gas environment is Ar, which flows downward onto the sample at approximately 0.5 m/s.

The emissivity measurements (directional effective band-limited emissivity at 850 nm with half-height width of 40 nm) result in three distinct values based on the local surface topography stemming from the laser scan history on the high-purity nickel sample. An emissivity of approximately 0.39 is measured on the virgin surface. An emissivity of 0.36, with standard relative uncertainty of approximately 8%, is observed near the solidification temperature of the molten high-purity nickel, which is in good agreement with published values. Finally, an emissivity of approximately 0.33 is measured on the solidified melt tracks, due to the reduction in surface roughness caused by melting, followed by solidification.

The solidification temperature of high-purity nickel is measured to be 1 500 °C with a standard uncertainty of approximately 16 °C, which locates the expected solidification temperature of 1 455 °C outside of the bounds of the standard (*k* = 1) and expanded (*k* = 2) uncertainties. The emitted, scattered, and/or reflected radiation from the plume of the process by-products above the melt pool is currently believed to be the cause of the temperature measurement discrepancy, and future efforts may be directed toward rectification of the discrepancy. Nevertheless, the discrepancy between the measured solidification temperature and expected solidification temperature of only 45 °C (a 2.6% discrepancy in terms of absolute temperature) is among the most accurate temperature measurements of an LPBF melt pool yet reported in the open literature.

### 7.2 Future Efforts

Future efforts may include the implementation of the full range of temperature measurements by including different SSs. Future tests will also be performed on different materials and with varying processing parameters. Furthermore, the measurement uncertainties will be further evaluated, as described in Sec. 7.2.1. The test uncertainties required for established reference data based on the methods described here are discussed in Sec. 7.2.2.

#### 7.2.1 Sources of Measurement Uncertainties That May Require Further Evaluation

While the validation using the solidification of a high-purity metal has demonstrated very good agreement of the measurement results with published data, this does not necessarily indicate that all sources of uncertainty have received a comprehensive treatment. This is because some of them may be specific to the material, or they may become more significant away from the solidification plateau where the validation was performed. Furthermore, uncorrected sources of bias can potentially compensate for each other.

The first improvement to be made will be in the data analysis. In this paper, five pixels around the saturated area were rejected because of the deconvolution of erroneous saturated signal values. Moving forward, the signal values around the saturated region will either be extrapolated, or data at shorter integration times will be scaled and stitched into the saturated region to reduce the deconvolution error and reduce the number of rejected pixels.

A second improvement that will be implemented will be a calibration of the TISS850 with the Photron FastCAM Mini AX200 (CMOS FPA), which will eliminate the uncertainty due to the spectral mismatch of the TISS850 calibration. The TSP850 calibration step in Sec. 4.2.3 will be replaced with the CMOS FPA, and the TISS850 will then be calibrated with the CMOS FPA instead of the TSP850. This change in the calibration sequence ensures that the TISS850 will generate signals equivalent to that of the HTBB at each radiance temperature on the CMOS FPA, thus reducing the calibration uncertainty.

Another group of uncertainties is associated with the effects of the LPBF process by-products on measurements of both the thermally emitted light and reflectance. Such effects may be sensitive to the process and environmental parameters. There are several possibilities for evaluating such effects, which should significantly improve our understanding of laser delivery losses and defocusing, the thermal emission contribution from the hot metal vapor, and the absorptance and scattering of thermal radiation of the sample or the reflectometer illumination by these phenomena.

Another, significantly smaller concern is the effect of the imperfect hemispherical illumination during reflectometry. Our “dome” illumination source differs from an ideal uniform radiator in two ways—through spatial nonuniformity across the radiating area, and through a lack of illumination at the high angles (approximately starting at 75° from the normal to the sample surface), as well as at the observation port location (commonly referred to as “port loss”). The nonuniformity of the radiating surface has been mapped and is included in the uncertainty budget. The effects of high-angle and port losses can be minimized through an already planned redesign of the dome and an introduction of a port loss evaluation technique, which are planned to be implemented at the next stage.

Several hardware improvements may be implemented in the future to improve the utility of the emissivity measurement approach described here. The current reflectometer configuration uses continuous illumination by high-intensity LEDs at 850 nm. The LEDs are modularized and can be changed if necessary, for measurement in other spectral bands. LEDs at shorter wavelengths will enable measurement of equivalent radiance temperatures of the reflectometer’s illumination (for example, up to 2 500 °C for a 405 nm band illumination with a target emissivity of 0.5). Future hardware improvements also include the use of pulsed narrow-band sources to extend the temperature range further, adding the ability to translate the reflectometer, and improving upon the shield gas arrangement to be able to work with metal powders for LPBF research.

Use of melting/solidification points of high-purity metals for comparison to the measured temperatures provides a relatively unambiguous benchmark. However, certain conditions of the LPBF scanning process, as well as methods for identifying the solidification point from surface temperature data, may incur errors in the presumed benchmark temperature. Common methods and apparatus for measuring reference melting/solidification temperatures, such as differential thermal analysis (DTA), use isothermal conditions and low heating/cooling rates (<10^−2^ °C/s), resulting in “equilibrium” solidification conditions. Extracting the solidification point from the instrument’s profiles may use a variety of calculation techniques involving identification of the inflection points, linear interpolation or extrapolation, and intersection of these lines [54]. However, LPBF melt pools generate exceedingly high cooling rates measured near the solidification point (>10^5^ °C/s), which can induce strong nonequilibrium solidification, which in turn can affect the real solidification temperature. Furthermore, the high thermal gradients of LPBF melt pools, in conjunction with the process dynamics, may make identification of local inflection points or intersections nonrepeatable. Further testing of rate-dependent effects on solidification will be conducted by varying laser scan parameters, and methods for calculating the solidification point from temperature profiles will be reviewed in the context of those methods used under equilibrium conditions to generate reference solidification temperatures.

#### 7.2.2 Toward Establishing the Reference Thermography Data

##### 7.2.2.1 Reference Data and Establishing Uncertainties of the Test

A primary intended use of the established capability is the generation of reference thermographic data, such that the data are representative of the process parameters and sample and can be reproduced at a third-party facility. Accordingly, establishment of the LPBF temperature distribution sensitivity to the process and environmental parameters will enable accurate description of the conditions under which the data are taken, and the ability to predict the effects of uncertainties in these conditions during replication of the LPBF process at other machines.

##### 7.2.2.2 Laser-Related Test Parameters

Laser-related process parameters include laser power, motion velocity, spot diameter and its distribution, and uniformity of all these parameters across the nominal build plane. There are indications of thermal effects that may also change these parameters over time due to heating of the custom AMMT components, which may add extra complexity. It is anticipated that a commercial device will help to improve our understanding of all of the above matters.

##### 7.2.2.3 Sample-Related Test Parameters

Sample-related parameters include its surface flatness and finish, and material composition, as well as alignment relative to the nominal build plane. Here, we anticipate a major improvement from a dedicated laser displacement line sensor.

##### 7.2.2.4 Environmental Conditions

Environmental conditions include optical and gas flow parameters. The gas flow velocity and velocity profile above the build plane are important for removal of the by-products, which can have highly detrimental effects on laser coupling, melt pool size, and process variability. The oxygen content is another parameter that requires both characterization of the uncertainties of its measurements, as well as determination of the thermographic data sensitivity to oxygen content. An illuminating dome enclosure upgrade is necessary to achieve a well-developed gas flow that can be properly characterized using anemometry. It is anticipated that a new setup with proper flow provisions will be implemented in the future.
